# Protocol to profile tumor and microenvironment from ovarian cancer patient samples and evaluate cell-based therapy using *in vitro* killing assays

**DOI:** 10.1016/j.xpro.2025.103742

**Published:** 2025-04-05

**Authors:** Yan-Ruide Li, Christopher J. Ochoa, Zibai Lyu, Yichen Zhu, Yuning Chen, Gabriella DiBernardo, Lauryn Ruegg, Sanaz Memarzadeh, Lili Yang

**Affiliations:** 1 Department of Microbiology, Immunology & Molecular Genetics, University of California, Los Angeles, Los Angeles, CA 90095, USA; 2 Department of Bioengineering, University of California, Los Angeles, Los Angeles, CA 90095, USA; 3 Department of Obstetrics and Gynecology, David Geffen School of Medicine, University of California, Los Angeles, Los Angeles, CA 90095, USA; 4 Molecular Biology Institute, University of California, Los Angeles, Los Angeles, CA 90095, USA; 5 Eli and Edythe Broad Center of Regenerative Medicine and Stem Cell Research, University of California, Los Angeles, Los Angeles, CA 90095, USA; 6 Jonsson Comprehensive Cancer Center, David Geffen School of Medicine, University of California, Los Angeles, Los Angeles, CA 90095, USA; 7 The VA Greater Los Angeles Healthcare System, Los Angeles, CA 90073, USA; 8 Department of Medical and Molecular Pharmacology, University of California, Los Angeles, Los Angeles, CA 90095, USA; 9 Parker Institute for Cancer Immunotherapy, University of California, Los Angeles, Los Angeles, CA 90095, USA

**Keywords:** Cell-based Assays, Flow Cytometry, Cancer, Immunology

## Abstract

Ovarian cancer (OC) presents significant challenges due to late diagnosis and high recurrence rates, necessitating a deeper understanding of the molecular and cellular characteristics of OC and the exploration of novel therapeutic approaches. Here, we provide a protocol to characterize primary OC patient samples, including the tumor and the tumor microenvironment (TME), using flow cytometry. Additionally, we detail the design and evaluation of various cell-based immunotherapies aimed at targeting primary OC tumor and the TME through *in vitro* killing assays.

For complete details on the use and execution of this protocol, please refer to Li et al.^1^

## Highlights

• Profiling tumor and microenvironment using samples from ovarian cancer patients
• Generating therapeutic cells for ovarian cancer, including CAR-T, ESO-T, and NKT cells
• Assessing the efficacy of cell-based therapies targeting ovarian cancer tumor cells
• Studying NKT cell-based therapies targeting the ovarian cancer tumor microenvironment

## Abstract (editor highlights)

Publisher’s note: Undertaking any experimental protocol requires adherence to local institutional guidelines for laboratory safety and ethics.

## Graphical abstract

**Figure:**
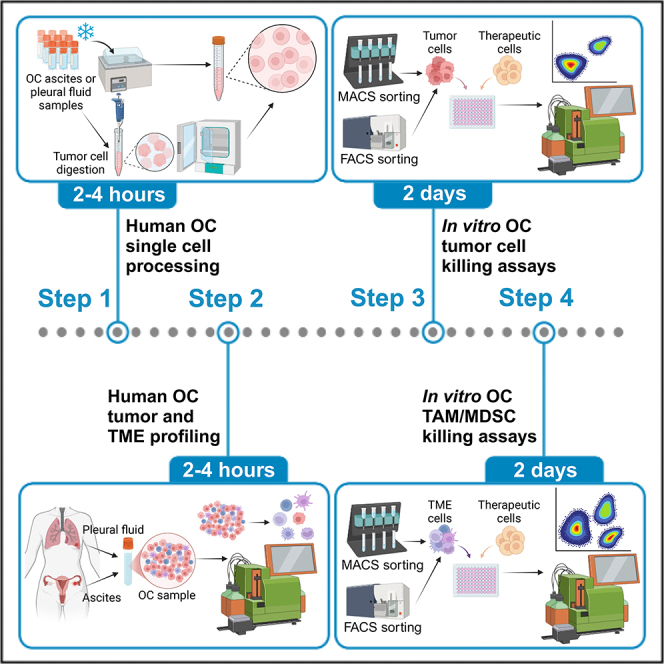

## Before you begin

The protocol below describes the specific procedures for the collection and processing of OC from pleural fluid or ascitic fluid derived from primary OC patients. This method facilitates the profiling of OC tumors and their TME. Additionally, the protocol encompasses the design and execution of *in vitro* killing assays aimed at targeting tumor cells and the TME through cell-based therapies.

The described methodology is adaptable for the profiling of other cancer types, particularly solid tumors. The *in vitro* killing assays provided can effectively target tumor cells and the TME, specifically focusing on immunosuppressive tumor-associated macrophages (TAMs) and myeloid-derived suppressor cells (MDSCs) present in various solid tumors.^2^^,^^3^^,^^4^^,^^5^ Importantly, the protocol includes techniques for generating chimeric antigen receptor (CAR)-engineered T (CAR-T) cells, NY-ESO-1 specific TCR engineered T (ESO-T) cells, and invariant natural killer T (NKT) cells. These methodologies serve as a guide for researchers aiming to develop cell-based immunotherapies tailored for various therapeutic purposes.

***Note:*** In addition to the cell-based immunotherapies previously mentioned, there are numerous other approaches for targeting OC and other solid tumors, including CAR-engineered natural killer (CAR-NK) cells,^6^^,^^7^ CAR-engineered macrophages,^8^ and CAR-engineered gamma delta (CAR-γδ) T cells.^9^ In this study, we focus primarily on evaluating the cell types that we have successfully utilized in prior experiments. However, our *in vitro* killing assays are versatile and can be adapted for testing additional cellular therapies, provided that appropriate modifications are made to the protocols.

### Institutional permissions

Healthy donor human peripheral blood-derived mononuclear cells (PBMCs) were obtained from the UCLA/CFAR Virology Core Laboratory, without identification information under federal and state regulations. The collection of primary OC patient samples was approved by the UCLA Office of the Human Research Protection Program, IRB #10-0727 and IRB #20-1659. Primary OC patient samples were collected from consented patients through an IRB-approved protocol (IRB #10-0727) and processed. Researchers intending to replicate this protocol must ensure they obtain the necessary permissions and approvals from their respective institutional committees.

### Human OC processing and cryopreservation

**Timing: 3 h**

**Figure 1:**
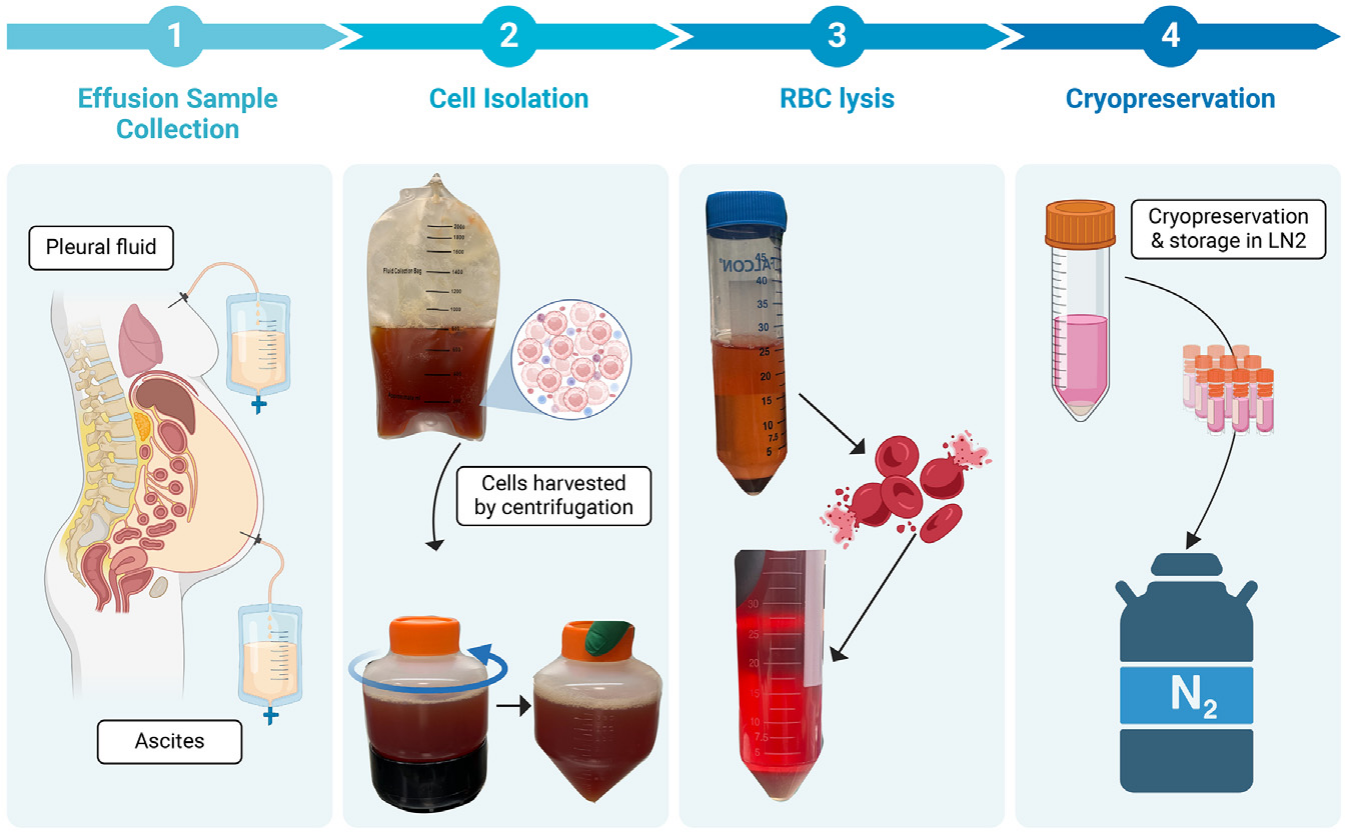
Primary OC patient sample collection and processing

1. Retrieve and process ascites or pleural fluid samples immediately (Figure 1).
  a. Pour the ascites or pleural fluid sample into 50-mL conical tubes or 500-mL centrifuge tubes depending on volume.
    **CRITICAL:** Begin processing of ascites or pleural fluid sample as soon as possible to minimize cell death. Processing the samples within three hours of collection typically ensures a high percentage of viable cells.
  b. Centrifuge the sample at 500 x g for 5 min.
    ***Note:*** In our protocol, centrifugation is performed to pellet the cells, including OC tumor cells, TME cells, and therapeutic cells. Conducting the centrifugation at 4°C helps to maintain a high viability of live cells; therefore, it is recommended to carry out the centrifugation at this temperature.
  c. Carefully aspirate supernatant.
  d. Repeat above steps, 1a-1c, until all of the sample is spun down. For large volumes, the samples can be kept on ice during the process.
  e. If red blood cells (RBCs) are present, perform an RBC lysis of cell pellet. Incubate the cell pellet with lysis buffer for 15 min at 20°C–25°C.
  f. Centrifuge at 500 x g for 5 min after RBC lysis and resuspend in cold RPMI media without supplements.
  g. Count the number of cells and check viability.
    ***Note:*** Tumor cells from ascites or pleural fluid samples may be in an aggregated, spheroid form and getting an accurate cell count may be difficult. If this is the case, getting an estimate will suffice.
  h. Centrifuge the ascites or pleural fluid sample at 500 x g for 5 min.
  i. Resuspend in cold sterile cryopreservation buffer, consisting of FBS with 10% DMSO.
    **CRITICAL:** During cryopreservation process, keep sample on ice and work quickly to freeze down. Serological pipettes may be kept in freezer prior to cryopreservation.
  j. Aliquot sample into cryopreservation tubes on ice.
    ***Note:*** Limit number of total concentration of cells cryopreserved to a maximum of 1 × 10^7^ cells/mL per vial. If tumor cells are heavily aggregated, getting an estimate for this step will suffice.

### Human CAR-T cell generation

**Timing: 2 weeks**

**Figure 2:**
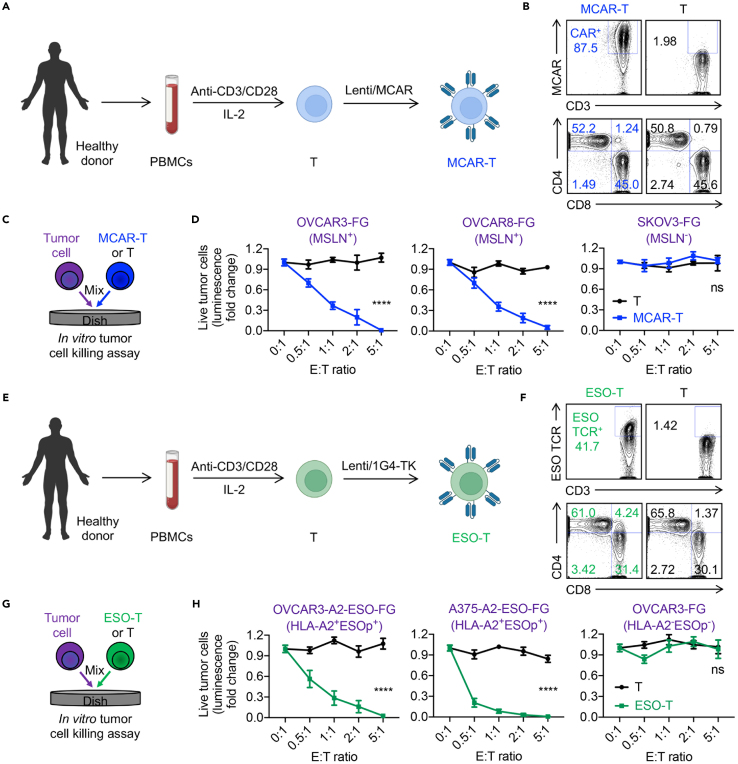
Generation and tumor cell killing capacity of human PBMC-derived MCAR-T and ESO-T cells (A and B) Generation of healthy donor PBMC-derived MCAR-T cells. (A) Experimental design. (B) FACS analyses of MCAR and CD4/CD8 co-receptor expressions on MCAR-T cells. (C and D) Evaluation of *in vitro* tumor cell killing efficacy of MCAR-T cells. (C) Experimental design. (D) Tumor cell killing data of MCAR-T cells at 24 h (*n* = 4). (E and F) Generation of healthy donor PBMC-derived ESO-T cells. (E) Experimental design. (F) FACS analyses of ESO TCR and CD4/CD8 co-receptor expressions on ESO-T cells. (G and H) Evaluation of *in vitro* tumor cell killing efficacy of ESO-T cells. (G) Experimental design. (H) Tumor killing data of ESO-T cells at 24 h (*n* = 4). Representative of > 5 experiments. Data are presented as the mean ± SEM. ns, not significant, ∗∗∗∗*p* < 0.0001, by Student’s *t* test (D and H).
3. Incubate coated plate at 37°C cell culture incubator for 1.5 h or 4°C for 18–24 h.
4. Seed human PBMCs in the coated plate.
  a. Aspirate coating solution from each well and wash with 1 x PBS buffer.
  b. Thaw the healthy donor-derived PBMCs from liquid nitrogen in a 37°C water bath. Carefully add the thawed cells to C10 medium dropwise.
  c. Centrifuge the cells at 300 x g for 10 min, and then carefully aspirate the supernatant.
  d. Resuspend the PBMCs in C10 medium supplemented with 30 ng/mL recombinant human IL-2 and 2 μg/mL anti-human CD28 antibody at a density of 1 × 10^6^ cells/mL.
  e. Dispense 2 mL of the cell suspension into each well of the 24-well plate.
5. Culture the cells in a humidified CO_2_ incubator at 37°C for 48 h.
6. At Day 2, thaw and add 20 μL concentrated Lenti/MCAR virus supernatant to each well containing 2 mL of cell culture.
  ***Note:*** Using the anti-human CD3 antibody will specifically activate and transduce only the human CD3^+^ T cells with lentivirus, leading to the eventual death of other cell types, such as B cells, NK cells, and monocytes. As a result, the final purity of T cells will be enriched to over 95%.
  ***Note:*** MCAR refers to a mesothelin-targeting CAR using a third-generation CAR design that includes both CD28 and 4-1BB transmembrane domains.^10^
  ***Note:*** The protocol of generating lentivirus is described in previous papers.^11^^,^^12^
  a. Gently pipette the thawed supernatant to mix (avoid vortexing) and add it directly to the wells.
  b. Gently rock the plate to ensure mixing.
7. Culture the cells in a humidified CO_2_ incubator at 37°C for two weeks.
  a. When the cell density exceeds 2.5 × 10^6^ cells/mL or when the medium turns yellow, split cultures back to a density of 0.5–1 × 10^6^ cells/mL in culture medium containing 10 ng/mL recombinant human IL-2.

***Note:*** Monitor the cell culture and check the cell morphology daily. Cell shrinkage and a reduced proliferation rate typically indicate exhausted cultures.

***Note:*** This protocol outlines the preparation of mesothelin-targeting CAR-engineered T (MCAR-T) cells derived from healthy donor PBMCs (Figure 2A). The resulting MCAR-T cells were utilized to assess their cytotoxicity against primary OC tumor cells.

2. On Day 0, prepare 2 μg/mL anti-human CD3 antibody solution in sterile 1 x PBS buffer.
  a. Calculate the number of wells required for each experimental condition. For one 24-well plate, 6 mL of antibody solution is needed.
  b. Add 250 μg of the antibody solution into each well of the 24-well plate.

8. Collect all the cell culture and centrifuge at 300 x g for 10 min. Carefully aspirate the supernatant.
9. Freeze down the generated MCAR-T cells in Cryostor Cell Cryopreservation Media at a concentration of 1 × 10^7^ cells/mL.
10. Use flow cytometry to evaluate the MCAR expression using a Goat anti-Mouse IgG F(ab’)2 Secondary Antibody on MCAR-T cells (Figure 2B).

**CRITICAL:** Ensure that MCAR expression exceeds 50% on the MCAR-T cells for successful downstream analysis.

11. Perform *in vitro* tumor cell killing assays to assess the functionality of the MCAR-T cells (Figures 2C and 2D).

### Human ESO-T cell generation

**Timing: 2 weeks**

***Note:*** This protocol outlines the preparation of ESO-T cells derived from healthy donor PBMCs (Figure 2E). The resulting ESO-T cells were utilized to assess their cytotoxicity against primary OC tumor cells.

12. On Day 0, prepare 2 μg/mL anti-human CD3 antibody solution in sterile 1 x PBS buffer.
  a. Calculate the number of wells required for each experimental condition. For one 24-well plate, 6 mL of antibody solution is needed.
  b. Add 250 μL of the antibody solution into each well of the 24-well plate.
13. Incubate coated plate at 37°C for 1.5 h or 4°C for 18–24 h.
14. Seed human PBMCs in the coated plate. The procedure is similar to that described for generating MCAR-T cells mentioned above.
  a. Aspirate coating solution from wells and wash with 1 x PBS buffer.
  b. Prepare single cell suspension of PBMCs in C10 medium supplemented with 30 ng/mL recombinant human IL-2 and 2 μg/mL anti-human CD28 antibody at a density of 1 × 10^6^ cells/mL.
  c. Dispense 2 mL of the cell suspension into each well of the 24-well plate.
15. Culture the cells in a humidified CO_2_ incubator at 37°C for 48 h.
16. At Day 2, thaw and add 20 μL concentrated Lenti/1G4-TK virus supernatant to each well containing 2 mL of cell culture.
  ***Note:*** The 1G4 receptor represents an HLA-A2-restricted ESO TCR, while the TK gene serves as a suicide mechanism, functioning as a safety switch to enhance therapeutic control.
  a. Gently pipette the thawed supernatant to mix (avoid vortexing) and add it directly to the wells.
  b. Gently rock the plate to ensure mixing.
17. Culture the cells in a humidified CO_2_ incubator at 37°C for two weeks.
  a. When the cell density exceeds 2.5 × 10^6^ cells/mL or when the medium turns yellow, split cultures back to a density of 0.5–1 × 10^6^ cells/mL in culture medium containing 10 ng/mL IL-2.

***Note:*** Monitor the cell culture and check the cell morphology daily. Cell shrinkage and a reduced proliferation rate typically indicate exhausted cultures.

18. Collect all the cell culture and centrifuge at 300 x g for 10 min. Carefully aspirate the supernatant.
19. Freeze down the generated ESO-T cells in Cryostor Cell Cryopreservation Media at a concentration of 1 × 10^7^ cells/mL.
20. Use flow cytometry to evaluate the ESO TCR expression using an anti-human TCRVβ13.1 antibody on ESO-T cells (Figure 2F).

**CRITICAL:** Ensure that ESO TCR expression exceeds 50% on the ESO-T cells for successful downstream analysis.

21. Perform *in vitro* tumor cell killing assays to assess the functionality of the ESO-T cells (Figures 2C and 2D).

### Human NKT cell generation

**Timing: 6 weeks**

**Figure 3:**
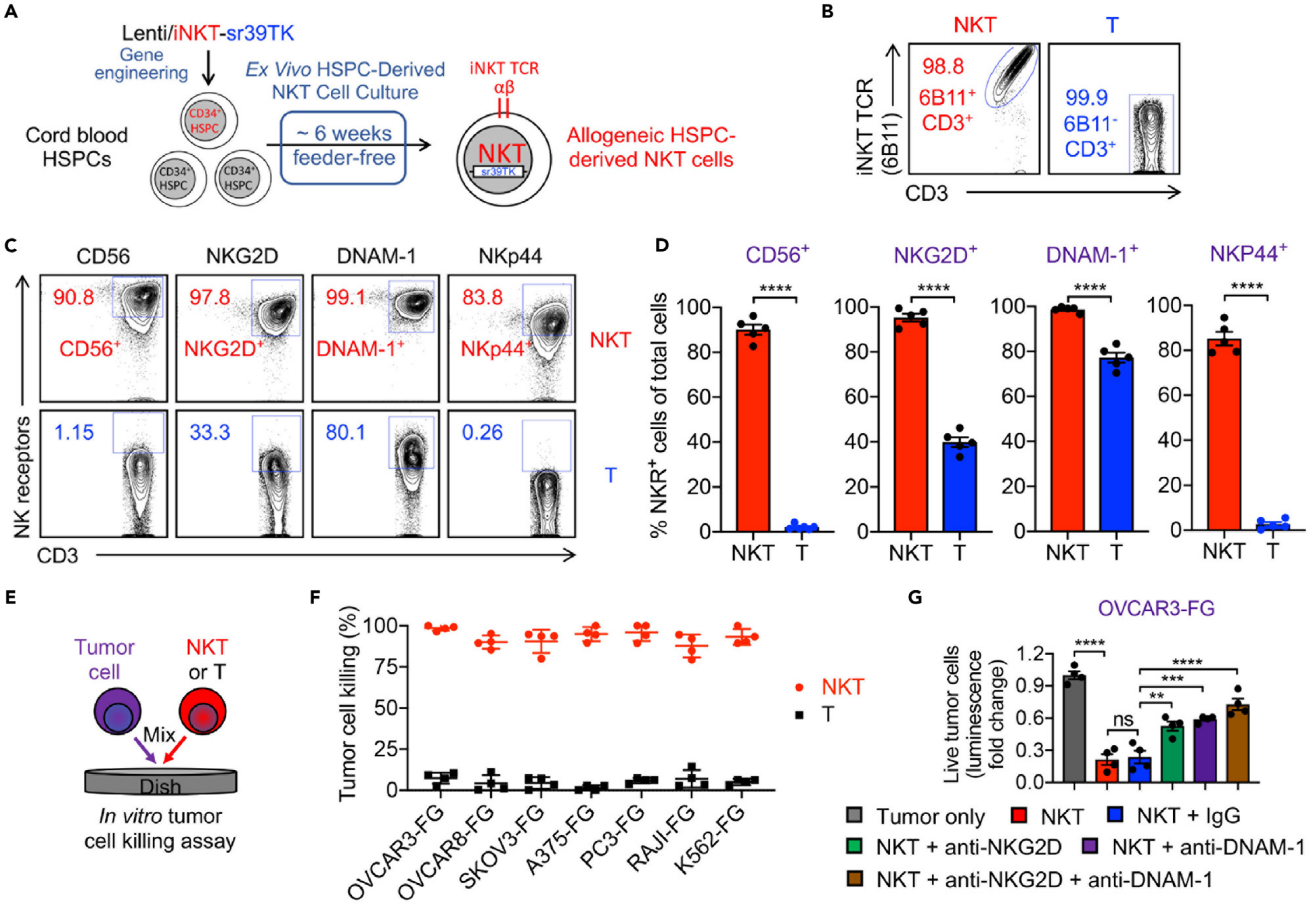
Generation and tumor cell killing capacity of human HSPC-derived NKT cells (A–D) Generation and characterization of human HSPC-derived NKT cells. (A) Experimental design. (B) FACS analyses of NKT TCR expression on NKT cell. (C) FACS analyses of NK marker expression on NKT cells. (D) Quantification of (C) (*n* = 5). (E and F) Studying *in vitro* tumor cell killing efficacy of NKT cells. (E) Experimental design. Healthy donor PBMC-derived conventional T cells are included as a therapeutic cell control. (F) Tumor cell killing data of NKT cells at 24 h (*n* = 4). Seven human tumor cell lines are utilized. (G) Studying *in vitro* tumor cell killing mechanism of NKT cells mediated by NK activating receptors (i.e., NKG2D and DNAM-1). Tumor cell killing data at 24 h are presented (*n* = 4). Representative of > 5 experiments. Data are presented as the mean ± SEM. ns, not significant, ∗∗*p* < 0.01, ∗∗∗*p* < 0.001, ∗∗∗∗*p* < 0.0001, by Student’s *t* test (D) or one-way ANOVA (G).
23. Aspirate the RN solution and replace it with 1 mL of 2% BSA in 1 x PBS buffer. Incubate the wells at 20°C–25°C for 30 min.
24. Aspirate the 2% BSA solution and replace it with 2 mL of 1 x PBS buffer.

***Note:*** Use the RN-coated plate on the same day to ensure optimal coating efficiency.

***Note:*** This protocol outlines the preparation of NKT cells derived from human cord blood CD34^+^ hematopoietic stem and progenitor cells (HSPCs) (Figure 3A). The resulting NKT cells were utilized to assess their cytotoxicity against primary OC tumor cells and their TME, specifically targeting TAMs and MDSCs within the TME.

22. On Day -2, coat 6-well non-tissue culture-treated plate using retronectin (RN).
  a. Thaw the RN stock stored at −20°C. Dilute the RN to 20 μg/mL in sterile 1 x PBS buffer.
  b. Dispense 1 mL of the diluted RN solution into each well.
  c. Incubate at 20°C–25°C for 2 h.

25. Thaw human cord blood-derived CD34^+^ HSPCs using the X-VIVO 15 serum-free hematopoietic stem cell medium.
26. Centrifuge the cells at 300 x g for 10 min.
27. Aspirate the supernatant and resuspend the cell pellet in X-VIVO 15-based HSPC culture medium.
28. Count the cells to determine the total cell concentration.

***Note:*** HSPC culture medium is prepared by supplementing the X-VIVO 15 medium with 50 ng/mL recombinant human SCF, 50 ng/mL recombinant human Flt3 ligand, 50 ng/mL recombinant human thrombopoietin (TPO) and 20 ng/mL recombinant human IL-3.

29. Centrifuge the cells again at 300 x g for 10 min.
30. Aspirate the supernatant and resuspend the CD34^+^ HSPCs at a final concentration of 1 × 10^6^ cells/mL in HSPC culture medium.
31. Aspirate the 1 × PBS buffer from the RN-coated wells. Seed 1 mL of the HSPC suspension into each well of the coated 6-well plate.
32. Incubate in a humidified CO_2_ incubator at 37°C for 18–24 h.
33. On Day -1, thaw and add the concentrated Lenti/iNKT-sr39TK virus supernatant.
  ***Note:*** The sr39TK gene serves as a suicide mechanism, functioning as a safety switch to enhance therapeutic control.^11^
  a. Gently pipette the thawed supernatant to mix (avoid vortexing) and add it directly to the wells.
  b. Gently rock the plate to ensure mixing.
34. Culture the cells in a humidified CO_2_ incubator at 37°C for 18–24 h.
35. On Day 0, coat 24-well non-treated multiwell plate with the StemSpan Lymphoid Differentiation Coating Material (100X).
  a. Dispense 500 μL of the diluted coating material into each well.
  b. Coat the plate at 20°C–25°C for 2 h or at 4°C for 18–24 h.
36. Collect the cells after incubation by gently pipetting.

***Note:*** Wash the wells with an equal volume of cold HSPC culture medium to recover any remaining adherent cells. Repeat this step as necessary to ensure all cells are collected.

37. Centrifuge the collected cells at 300 x g for 10 min.
38. Aspirate the supernatant and resuspend the cell pellet in stage 0 HSPC culture medium.
39. Count the cells to determine the total cell concentration.
40. Centrifuge the cells at 300 x g again for 10 min.
41. Aspirate the supernatant and resuspend the cells at a final concentration of 2 × 10^4^ cells/500 μL in HSPC expansion medium.

***Note:*** HSPC expansion medium is prepared by supplementing StemSpan SFEM II Medium with 10× StemSpan Lymphoid Progenitor Expansion Supplement.

42. Culture the cells in a humidified CO_2_ incubator at 37°C for 3 days.
43. On Day 4, supplement each well with an additional 500 μL of fresh HSPC expansion medium. Continue to culture the cells for an additional 4 days.

**CRITICAL:** Monitor cell density daily. If overgrowth occurs (e.g., >70% confluence or yellowing of the medium), split the cells by collecting, centrifuging, and resuspending them in fresh medium. Seed the cells into two separate wells to reduce density.

44. On Day 7, replace half of the old medium with 500 μL of fresh HSPC expansion medium. Repeat this step on Day 11 and continue to culture the cells for an additional 3 days.
45. On Day 14, coat 6-well non-tissue culture-treated plate with the StemSpan Lymphoid Differentiation Coating Material (100X).
  a. Dispense 1.5 mL of the coating material into each well.
  b. Coat the plate at 20°C–25°C for 2 h or at 4°C for 18–24 h.
46. Collect cells from previous stage, centrifuge at 300 x g for 10 min, and resuspend them in NKT differentiation medium at a final concentration of 5 × 10^5^ cells/mL.

***Note:*** Optionally, cryopreserve any remaining cells at a concentration of 2 × 10^5^ cells/500 μL in Cryostor Cell Cryopreservation Media. Cryopreserved cells can be stored in liquid nitrogen for up to 6 months.

***Note:*** NKT differentiation medium is prepared by supplementing StemSpan SFEM II medium with 10× StemSpan Lymphoid Progenitor Maturation Supplement.

47. Seed 2 mL of the cell suspension into each coated well and culture at 37°C in a 5% CO_2_ incubator for 7 days.

***Note:*** During this time, passage the cells 2–3 times to maintain a density of 0.5–1 × 10^6^ cells/mL.

**CRITICAL:** If overgrowth occurs (e.g., > 70% confluence or yellowing of the medium), split the cells by collecting, centrifuging, and resuspending them in fresh medium. Seed the cells into two separate wells to reduce density.

48. On Day 21, coat 6-well non-tissue culture-treated plate with the StemSpan Lymphoid Differentiation Coating Material (100X).
  a. Dispense 1.5 mL of the coating material into each well.
  b. Coat the plate at 20°C–25°C for 2 h or at 4°C for 18–24 h.

***Note:*** At the end of the differentiation period, collect a small portion of cells (∼1 × 10^5^ cells) and analyze using flow cytometry. Stain with markers for NKT differentiation, including NKT TCR, CD3, TCRαβ, CD4, CD8, and a cell viability dye (e.g., e506).

49. Collect cells from the previous stage, centrifuge at 300 x g for 10 min, and resuspend them in NKT deep differentiation medium at a final concentration of 5 × 10^5^ cells/mL.

***Note:*** NKT deep differentiation medium is prepared by supplementing StemSpan SFEM II medium with 10 × StemSpan Lymphoid Progenitor Maturation Supplement, 10 ng/mL recombinant human IL-15, and 12.5 μL/mL ImmunoCult human CD3/CD28/CD2 T cell activator.

50. Seed 2 mL of the cell suspension into each coated well and culture at 37°C in a 5% CO_2_ incubator for 7 days.

***Note:*** During this period, passage the cells 2–3 times to maintain a density of 0.5–1 × 10^6^ cells/mL.

**CRITICAL:** If overgrowth occurs (e.g., >70% confluence or yellowing of the medium), split the cells by collecting, centrifuging, and resuspending them in fresh medium. Seed the cells into two separate wells to reduce density.

51. On Day 28, select one of three expansion methods: αCD3/αCD28 antibody-based expansion, α-Galactosylceramide-loaded PBMC (αGC/PBMC) co-culture expansion, or artificial antigen presenting cell (aAPC) co-culture expansion.
  ***Note:*** At the end of the deep differentiation stage, collect a small portion of cells (∼1 × 10^5^ cells) and analyze via flow cytometry. Stain with markers for NKT deep differentiation, including NKT TCR, CD3, TCRαβ, CD4, CD8, and a viability dye. Expect > 95% of cells to be NKT TCR^+^CD3^+^TCRαβ^+^ cells.
  **CRITICAL:** For all methods, maintain a culture density of 0.5–1 × 10^6^ cells/mL throughout the expansion phase.
  ***Note:*** The NKT expansion medium is prepared by supplementing C10 medium with 10 ng/mL recombinant human IL-7 and 10 ng/mL recombinant human IL-15.
  ***Note:*** For all methods, passage cells 2–3 times/week in NKT expansion medium.
  For αCD3/αCD28 antibody expansion:
  a. Coat wells with 2 μg/mL anti-human CD3 antibody in 1 x PBS buffer and incubate at 20°C–25°C for 2 h or at 4°C for 18–24 h. Wash the well using 1 mL of 1 x PBS buffer.
  b. Resuspend NKT cells in NKT expansion medium with an additional 2 μg/mL of anti-human CD28 antibody.
  c. Seed 2 mL of the co-culture into each well of a 6 well plate and incubate for 2 weeks.
    For αGC/PBMC co-culture expansion:
  d. Load healthy donor PBMCs with 5 μg/mL αGC in C10 medium, irradiate at 6,000 rads, and co-culture with NKT cells at an NKT:PBMC ratio of 1:2–1:5.
  e. Resuspend NKT cells in NKT expansion medium and seed 2 mL of the co-culture into each well of a 6-well plate.
  f. Incubate for 2 weeks.
    For aAPC co-culture expansion:
  g. Irradiate aAPCs at 10,000 rads, filter to remove aggregates, and co-culture with NKT cells at an NKT:aAPC ratio of 1:1–1:2.
  h. Resuspend NKT cells in NKT expansion medium and seed 2 mL of the co-culture into each well of a 6-well plate.
52. At the end of the 2-week expansion phase, freeze the NKT cells in Cryostor Cell Cryopreservation Media at a concentration of 1 × 10^7^ cells/mL.
53. Use flow cytometry to evaluate the transgenic NKT TCR expression (anti-human TCR Va24-Jb18) on NKT cells.

**CRITICAL:** NKT TCR expression should be > 95% on the NKT cells (Figure 3B).

***Note:*** Flow cytometry should be used to analyze the surface markers on NKT cells, including NK receptors such as CD56, NKG2D, DNAM-1, and NKp44 (Figures 3C and 3D).

54. Perform *in vitro* tumor cell killing assays to assess the functionality of the NKT cells, using various tumor cell lines (Figures 3E–3G).

### Human tumor cell line engineering

**Timing: 3 days**

***Note:*** This protocol outlines the development of a series of human tumor cell lines designed for use in *in vitro* tumor cell killing assays to assess therapeutic cell efficacy. It can be adapted for a wide range of tumor cell lines beyond human OC cell lines.

55. Culture tumor cell lines (e.g., Raji, K562, A375, OVCAR3, OVCAR8, SKOV3, or PC3) in R10 medium.
56. Seed 0.5–1 × 10^6^ tumor cells into one well of a 6-well plate.
57. Thaw and add the concentrated Lenti/FG, Lenti/HLA-A2 or Lenti/ESOp virus supernatant.
  ***Note:*** HLA-A2 refers to human HLA-A2, and ESOp refers to NY-ESO-1(157–165) peptide. The tumor cells engineered with both HLA-A2 and ESOp can be recognized by human ESO-T cells.^13^ FG denotes firefly luciferase and enhanced green fluorescent protein dual-reporters. The tumor cells engineered with FG can be monitored using flow cytometry and luminescence assays.^13^
  a. Gently pipette the thawed supernatant to mix (avoid vortexing) and add it directly to the wells.
  b. Gently rock the plate to ensure mixing.
  c. After 16–24 h of incubation, add another 1 mL of R10 medium to avoid tumor cell death.
58. Culture the cells in a humidified CO_2_ incubator at 37°C for 3 days. Regularly split the tumor cells to prevent overgrowth.
59. Confirm HLA-A2 and ESOp expression on tumor cell lines using flow cytometry. Confirm FG expression using flow cytometry or fluorescence microscopy.

***Note:*** This step should be performed three days after virus transduction to allow sufficient time for the tumor cells to express the transduced gene and transport the resulting protein to the cell membrane.

***Note:*** FACS sorting may be used to ensure 100% expression of the overexpressed genes.

60. Freeze down the engineered tumor cells in Cryostor Cell Cryopreservation Media at a concentration of 1 × 10^7^ cells/mL.

***Note:*** The materials and equipment section provides comprehensive recipes for all solutions, while the step-by-step method details section outlines the detailed procedures mentioned earlier. Prepare solutions beforehand, if possible, except for cell culture solutions. The cell culture media should be prepared fresh to ensure optimal cell growth and viability. Please refer to the key resources table for a comprehensive list of materials and equipment.

61. Culture the cells in a humidified CO_2_ incubator at 37°C for 3 days. Regularly split the tumor cells to prevent overgrowth.

***Note:*** Monitor the cell culture and check the cell morphology daily. When the plate becomes confluent, split the tumor cells to two or three new plates.

62. Confirm HLA-A2 and ESOp expression on tumor cell lines using flow cytometry. Confirm FG expression using flow cytometry or fluorescence microscopy.

***Note:*** This step should be performed three days after virus transduction to allow sufficient time for the tumor cells to express the transduced gene and transport the resulting protein to the cell membrane.

***Note:*** FACS sorting may be used to ensure 100% expression of the overexpressed genes.

63. Freeze down the engineered tumor cells in Cryostor Cell Cryopreservation Media at a concentration of 1 × 10^7^ cells/mL.

***Note:*** The materials and equipment section provides comprehensive recipes for all solutions, while the step-by-step method details section outlines the detailed procedures mentioned earlier. Prepare solutions beforehand, if possible, except for cell culture solutions. The cell culture media should be prepared fresh to ensure optimal cell growth and viability. Please refer to the key resources table for a comprehensive list of materials and equipment.

## Key resources table

**Table:** 

REAGENT or RESOURCE	SOURCE	IDENTIFIER
**Antibodies**		

Anti-human TCR αβ (clone I26, 1:25 dilution)	BioLegend	CAT#306716, RRID: AB_1953257
Anti-human TCR Vβ13.1 (clone H131, 1:200 dilution)	BioLegend	CAT#362403, RRID: AB_2564031
Anti-human CD1d (clone 51.1, 1:50 dilution)	BioLegend	CAT#350308, RRID: AB_10642829
Anti-human CD3 (clone HIT3a, 1:500 dilution)	BioLegend	CAT#300329, RRID: AB_10551436
Anti-human CD4 (clone OKT4, 1:400 dilution)	BioLegend	CAT#317414, RRID: AB_571959
Anti-human CD8 (clone SK1, 1:300 dilution)	BioLegend	CAT#344714, RRID: AB_2044006
Anti-human CD11b (clone ICRF44, 1:500 dilution)	BioLegend	CAT#301330, RRID: AB_2561703
Anti-human CD14 (clone HCD14, 1:100 dilution)	BioLegend	CAT#325608, RRID: AB_830681
Anti-human CD19 (clone SJ25C1, 1:100 dilution)	BioLegend	CAT#363005, RRID: AB_2564127
Anti-human CD24 (clone ML5, 1:200 dilution)	BioLegend	CAT#311113, RRID: AB_2561283
Anti-human CD31 (clone WM59, 1:100 dilution)	BioLegend	CAT#303117, RRID: AB_2114314
Anti-human CD34 (clone 581, 1:500 dilution)	BioLegend	CAT#343505, RRID: AB_1731937
Anti-human CD44 (clone BJ18, 1:50 dilution)	BioLegend	CAT#397503, RRID: AB_2814372
Anti-human CD45 (clone HI30, 1:500 dilution)	BioLegend	CAT#304026, RRID: AB_893337
Anti-human CD56 (clone HCD56, 1:10 dilution)	BioLegend	CAT#362545, RRID: AB_2565963
Anti-human CD86 (clone IT2.2, 1:1,000 dilution)	BioLegend	CAT#374209, RRID: AB_2728391
Anti-human CD112 (clone TX31, 1:50 dilution)	BioLegend	CAT#337409, RRID: AB_2174163
Anti-human CD117 (clone 104D2, 1:100 dilution)	BioLegend	CAT#313227, RRID: AB_2566214
Anti-human CD133 (clone clone7, 1:100 dilution)	BioLegend	CAT#372805, RRID: AB_2632881
Anti-human CD155 (clone SKII.4, 1:50 dilution)	BioLegend	CAT#337613, RRID: AB_2565746
Anti-human CD163 (clone GHI/61, 1:50 dilution)	BioLegend	CAT#333621, RRID: AB_2563611
Anti-human CD206 (clone 15-2, 1:100 dilution)	BioLegend	CAT#321110, RRID: AB_571885
Anti-human NKp30 (clone P30-15, 1:50 dilution)	BioLegend	CAT#325207, RRID: AB_756111
Anti-human NKp44 (clone P44-8, 1:50 dilution)	BioLegend	CAT#325107, RRID: AB_756099
Anti-human NKG2D (clone 1D11, 1:50 dilution)	BioLegend	CAT#320812, RRID: AB_2234394
Anti-human DNAM-1 (clone 11A8, 1:50 dilution)	BioLegend	CAT#338312, RRID: AB_2561952
Anti-human b2-microglobulin (B2M) (clone 2M2, 1:4,000 dilution)	BioLegend	CAT#316312, RRID: AB_10641281
Anti-human HLA-DR (clone L243, 1:250 dilution)	BioLegend	CAT#307618, RRID: AB_493586
Anti-human HLA-A2 (clone BB7.2, 1:500 dilution)	BioLegend	CAT#343310, RRID: AB_2561568
Anti-human MICA/MICB (clone 6D4, 1:100 dilution)	BioLegend	CAT#320908, RRID: AB_493195
Anti-human CTAG1B (W19067B, 1:200 dilution)	BioLegend	CAT#382302, RRID: AB_2922615
Anti-human CA125 (clone 618F, 1:100 dilution)	BioLegend	CAT#666904, RRID: AB_2629540
Anti-human HER2 (clone 24D2, 1:50 dilution)	BioLegend	CAT#324407, RRID: AB_756123
Anti-human EpCAM (clone 9C4, 1:50 dilution)	BioLegend	CAT#324203, RRID: AB_756077
Anti-human TROP2 (clone NY18, 1:50 dilution)	BioLegend	CAT#324203, RRID: AB_ 2572021
Anti-human FRα (clone No.5/FOLR, 1:50 dilution)	BioLegend	CAT#391805, RRID: AB_2721337
Anti-SOX2 (clone 14A6A34, 1:50 dilution)	BioLegend	CAT#656112, RRID: AB_2566189
Anti-Oct3/4 (clone 3A2A20, 1:50 dilution)	BioLegend	CAT#653704, RRID: AB_2562018
Human Fc receptor blocking solution (TrueStain FcX) (1:100 dilution)	BioLegend	CAT#422302, RRID: AB_2818986
Goat anti-mouse IgG (minimal x-reactivity) antibody (1:500 dilution)	BioLegend	CAT#405305, RRID: AB_315008
Goat anti-rat IgG (minimal x-reactivity) antibody (1:500 dilution)	BioLegend	CAT#405413, RRID: AB_10661733
Donkey anti-rabbit IgG (minimal x-reactivity) antibody (1:500 dilution)	BioLegend	CAT#406421, RRID: AB_2563484
Streptavidin (1:1,000 dilution)	BioLegend	CAT#405207
Anti-human TCR Va24-Jb18 (clone 6B11, 1:10 dilution)	BD Biosciences	CAT#552825, RRID: AB_394478
Anti-human mesothelin (MSLN) (clone 420411, 1:20 dilution)	R&D Systems	CAT#FAB32652P, RRID: AB_1151946
Anti-human fibroblast activation protein alpha/FAP (clone 427819, 1:100 dilution)	R&D Systems	CAT#MAB3715-SP
Anti-human ULBP-1 (clone 170818, 1:50 dilution)	R&D Systems	CAT#FAB1380P, RRID: AB_2687471
Anti-human ULBP-2,5,6 (clone 165903, 1:50 dilution)	R&D Systems	CAT#FAB1298A, RRID: AB_2257142
Goat anti-mouse IgG F(ab’)2 secondary antibody, biotin (1:50 dilution)	Thermo Fisher Scientific	CAT#31803, RRID: AB_228311
Anti-Nanog (clone EPR2027-2) (1:100 dilution)	Abcam	CAT#ab109250, RRID: AB_10863442

**Bacterial and virus strains**		

Lenti/FG	This paper	N/A
Lenti/HLA-A2	This paper	N/A
Lenti/ESOp	This paper	N/A
Lenti/iNKT-sr39TK	This paper	N/A
Lenti/MCAR	This paper	N/A
Lenti/1G4-TK	This paper	N/A

**Biological samples**		

Human peripheral blood mononuclear cells (PBMCs)	UCLA	N/A
Human cord blood CD34^+^ hematopoietic stem and progenitor cells (HSPCs)	Charles River	N/A
Human ovarian cancer patient samples	UCLA	N/A

**Chemicals, peptides, and recombinant proteins**		

Recombinant human IL-2	PeproTech	CAT#200–02
Recombinant human IL-3	PeproTech	CAT#200–03
Recombinant human IL-7	PeproTech	CAT#200–07
Recombinant human IL-15	PeproTech	CAT#200–15
Recombinant human Flt3-ligand	PeproTech	CAT#300–19
Recombinant human SCF	PeproTech	CAT#300–07
Recombinant human TPO	PeproTech	CAT#300–18
Recombinant human GM-CSF	PeproTech	CAT#300–03
L-ascorbic acid 2-phosphate	Sigma	CAT#A8960-5G
B-27 supplement (50X), serum-free	Thermo Fisher Scientific	CAT#17504044
α-galactosylceramide (KRN7000)	Avanti Polar Lipids	SKU#867000P-1mg
X-VIVO 15 serum-free hematopoietic cell medium	Lonza	CAT#04–418Q
UltraCULTURE media	Lonza	CAT#BP12725F
RPMI1640 cell culture medium	Corning Cellgro	CAT#10-040-CV
DMEM cell culture medium	Corning Cellgro	CAT#10-013-CV
CTS OpTmizer T cell expansion SFM	Gibco	CAT# A1048501
Fetal bovine serum (FBS)	Sigma	CAT#F2442
MACS BSA stock solution	Miltenyi	CAT#130-091-376
30% BSA	Gemini	CAT#700-110-100
Penicillin-streptomycin-glutamine (P/S/G)	Gibco	CAT#10378016
Penicillin:streptomycin (pen:strep) solution (P/S)	Gemini Bio-products	CAT#400–109
MEM non-essential amino acids (NEAA)	Thermo Fisher Scientific	CAT#11140050
HEPES buffer solution	Gibco	CAT#15630080
Sodium pyruvate	Gibco	CAT#11360070
Phosphate-buffered saline (PBS) pH 7.4 (1X)	Gibco	CAT#10010–023
Beta-mercaptoethanol	Sigma	SKU#M6250
Poloxamer Synperonic F108	Sigma	CAT#07579–250G-F
Normocin	InvivoGen	CAT#ant-nr-2
RetroNectin recombination human fibronectin fragment, 2.5 mg	Takara	CAT#T100B
Prostaglandin E2	Cayman Chemical	CAT#14-190-1
Collagenase type I	Thermo Fisher Scientific	CAT#17100017
Dispase II	Thermo Fisher Scientific	CAT#17105041
DNase I	Sigma-Aldrich	CAT#10104159001
Red blood cell (RBC) lysis buffer (10X)	Tonbo Biosciences	CAT#TNB-4300-L100
Dimethyl sulfoxide	Sigma	CAT#472301
Trypan blue solution, 0.4%	Thermo Fisher Scientific	CAT#15250061
Fixable viability dye eFluor506 Affymetrix	eBioscience	CAT#65-0866-14

**Critical commercial assays**		

Human CD34 MicroBeads kit	Miltenyi Biotec	CAT#130-046-703
Human CD14 MicroBeads kit	Miltenyi Biotec	CAT#130-050-201
Human CD45 MicroBeads kit	Miltenyi Biotec	CAT#130-045-801
Human anti-iNKT MicroBeads	Miltenyi Biotec	CAT#130-094-842
Human tumor cell isolation kit	Miltenyi Biotec	CAT#130-108-339
Fixation/permeabilization solution kit	BD Biosciences	CAT#55474
Foxp3/transcription factor staining buffer set	eBioscience	CAT#00-5523-00
StemSpan lymphoid differentiation coating material (100X)	STEMCELL Technologies	CAT#9925
StemSpan SFEM II	STEMCELL Technologies	CAT#9605
ImmunoCult human CD3/CD28/CD2 T cell activator	STEMCELL Technologies	CAT#10970
TransIT-Lenti transfection reagent	Mirus Bio	CAT#MIR 6600
Amicon Ultra-15 centrifugal filter unit	MilliporeSigma	CAT#UFC910024
CryoStor cell cryopreservation media	Sigma	CAT#C2874-100ML

**Experimental models: Cell lines**		

Human Burkitt’s lymphoma cell line RAJI	ATCC	CAT#CCL-86, RRID: CVCL_0511
Human Burkitt’s lymphoma cell line RAJI-FG	This paper	N/A
Human chronic myelogenous leukemia cell line K562	ATCC	CAT#CCL-243, RRID: CVCL_0004
Human chronic myelogenous leukemia cell line K562-FG	This paper	N/A
Human ovarian cancer cell line OVCAR3	ATCC	CAT#HTB-161, RRID: CVCL_0465
Human ovarian cancer cell line OVCAR3-FG	This paper	N/A
Human ovarian cancer cell line OVCAR3-A2-ESO-FG	This paper	N/A
Human ovarian cancer cell line SKOV3	ATCC	CAT#HTB-77, RRID: CVCL_0532
Human ovarian cancer cell line SKOV3-FG	This paper	N/A
Human prostate cancer cell line PC3	ATCC	CAT#CRL-1435, RRID: CVCL_0035
Human prostate cancer cell line PC3-FG	This paper	N/A
Human melanoma cell line A375	ATCC	CAT#CRL-1619, RRID: CVCL_0132
Human melanoma cell line A375-FG	This paper	N/A
Human melanoma cell line A375-A2-ESO-FG	This paper	N/A
Human ovarian cancer cell line OVCAR8	NIH	N/A
Human ovarian cancer cell line OVCAR8-FG	This paper	N/A

**Recombinant DNA**		

Vector: parental lentivector pMNDW	Giannoni et al.^14^; Lan et al.^15^	N/A

**Software and algorithms**		

FlowJo Software 9	FlowJo	https://www.flowjo.com/solutions/flowjo/downloads
Prism 8	GraphPad	https://www.graphpad.com/scientific-software/prism/

**Other**		

Branson M2800 mechanical ultrasonic cleaner	Marshall Scientific	CAT#CPX-952-216R
MACSQuant analyzer 10 flow cytometer	Miltenyi Biotec	CAT#130-096-343
Infinite M1000 microplate reader	Tecan	CAT#30190085
ChemiDoc touch gel imaging system	Bio-Rad	CAT#1708370
Drummond portable Pipet-Aid XP pipet controller	Fisher Scientific	CAT#13-681-06
Falcon 50 mL high clarity conical centrifuge tubes	Fisher Scientific	CAT#14-432-22
Corning 500 mL PP centrifuge tubes with plug seal cap, sterile	Corning	CAT#431123
Corning 500 mL polyetherimide centrifuge tube cushions	Corning	CAT#431124
Corning CoolCell FTS30 freezing container	Corning	CAT#CLS432008
Corning 2 mL internal threaded polypropylene cryogenic vial	Corning	CAT#430488
Steriflip-GP sterile centrifuge tube top filter unit	Millipore	CAT#SCGP00525
Sarstedt Inc. 1.5 mL screw cap micro tube, sterile	Fisher Scientific	CAT#50-809-238
Falcon 15 mL high clarity PP centrifuge tube, sterile	Corning	CAT#352097
Countess II automated cell counter	Thermo Fisher Scientific	CAT#AMQAX1000
Countess cell counting chamber slides	Thermo Fisher Scientific	CAT#C10312

## Materials and equipment

### C10 medium

• Prepare C10 medium for culturing T or NKT cells.
• Refer to the table below for the volumes needed to make 1 L C10 medium.
• Use an autoclaved 1-L bottle as the container for sterilized C10 medium.
• Attach a 0.22 μm filter top to the sterile bottle and filter the medium through.

**Table:** 

Reagent	Final concentration	Amount
RPMI	N/A	848 mL
FBS	10%	100 mL
Penicillin-Streptomycin-Glutamine (100X)	1X	10 mL
MEM NEAA (100X)	1X	10 mL
HEPES Buffer Solution (1 M)	0.01 M	10 mL
Sodium Pyruvate (100 mM)	1 mM	10 mL
β-ME (5 mM)	0.05 mM	10 mL
Normocin (500X)	1X	2 mL
**Total**	**N/A**	**1 L**

***Note:*** Store the C10 medium at 4°C. It is suitable for storage for up to one month.

### R10 medium

• Prepare R10 medium for culturing suspension tumor cells.
• Refer to the table below for the volumes needed to make 1 L R10 medium.
• Use an autoclaved 1-L bottle as the container for sterilized R10 medium.
• Attach a 0.22 μm filter top to the sterile bottle and filter the medium through.

**Table:** 

Reagent	Final concentration	Amount
RPMI	N/A	888 mL
FBS	10%	100 mL
Penicillin-Streptomycin-Glutamine (100X)	1X	10 mL
Normocin (500X)	1X	2 mL
**Total**	**N/A**	**1 L**

***Note:*** Store the R10 medium at 4°C. It is suitable for storage for up to one month.

### HSPC culture medium

• Prepare the HSPC Culture Medium for seeding human cord blood-derived CD34^+^ HSPCs.
• Refer to the table below for the volumes needed to make 50 mL HSPC culture medium.
• Attach a 0.22-μm filter top to the sterile bottle and filter the medium through.

**Table:** 

Reagent	Final concentration	Amount
X-VIVO-15 Serum-Free Hematopoietic Stem Cell Medium	N/A	49.83 mL
Human recombinant SCF	50 ng/mL	50 μL
Human recombinant Flt3L	50 ng/mL	50 μL
Human recombinant TPO	50 ng/mL	50 μL
Human recombinant IL-3	20 ng/mL	20 μL
**Total**	**N/A**	**50 mL**

***Note:*** The X-VIVO-15 medium stock should be stored at 4°C, protected from light.

***Note:*** Store the HSPC culture medium at 4°C. It is suitable for storage for up to one month.

### HSPC expansion medium

• Refer to the table below for the volumes needed to make 50 mL HSPC expansion medium.
• Attach a 0.22-μm filter top to the sterile bottle and filter the medium through.

**Table:** 

Reagent	Final concentration	Amount
StemSpan SFEM II Serum Free Medium	N/A	45 mL
StemSpan Lymphoid Progenitor Expansion Supplement (10X)	1X	5 mL
**Total**	**N/A**	**50 mL**

***Note:*** Freshly prepared medium remains viable at 4°C for ∼2 weeks. To ensure optimal quality and experimental integrity, discard any medium that exceeds this 2-week period.

### NKT differentiation medium

• Refer to the table below for the volumes needed to make 50 mL NKT differentiation medium.
• Attach a 0.22-μm filter top to the sterile bottle and filter the medium through.

**Table:** 

Reagent	Final concentration	Amount
StemSpan SFEM II Serum Free Medium	N/A	45 mL
StemSpan Lymphoid Progenitor Maturation Supplement (10X)	1X	5 mL
**Total**	**N/A**	**50 mL**

***Note:*** Freshly prepared medium remains viable at 4°C for ∼2 weeks. To ensure optimal quality and experimental integrity, discard any medium that exceeds this 2-week period.

### NKT deep differentiation medium

• Refer to the table below for the volumes needed to make 50 mL NKT deep differentiation medium.
• Attach a 0.22-μm filter top to the sterile bottle and filter the medium through.

**Table:** 

Reagent	Final concentration	Amount
StemSpan SFEM II Serum Free Medium	N/A	44.99 mL
StemSpan Lymphoid Progenitor Maturation Supplement (10X)	1X	5 mL
Human recombinant IL-15	10 ng/mL	10 μL
**Total**	**N/A**	**50 mL**

***Note:*** Freshly prepared medium remains viable at 4°C for ∼2 weeks. To ensure optimal quality and experimental integrity, discard any medium that exceeds this 2-week period.

### NKT expansion medium

• NKT expansion medium can be prepared using either i) a feeder-free, serum-free CTS OpTmizer T-Cell Expansion SFM or ii) a homemade C10 medium, supplemented with 10 ng/mL IL-7 and 10 ng/mL IL-15.
• Refer to the table below for the volumes needed to make 50 mL NKT expansion medium.
• Attach a 0.22-μm filter top to the sterile bottle and filter the medium.

**Table:** 

Reagent	Final concentration	Amount
CTS OpTmizer T-Cell Expansion SFM or C10 medium	N/A	49.98 mL
Human recombinant IL-7	10 ng/mL	10 μL
Human recombinant IL-15	10 ng/mL	10 μL
**Total**	**N/A**	**50 mL**

***Note:*** Freshly prepared medium remains viable at 4°C for ∼4 weeks. To ensure optimal quality and experimental integrity, discard any medium that exceeds this 2-week period.

### Working digestion mixture

• The working digestion mixture is prepared to enzymatically digest OC samples and generate single-cell suspensions.
• To create the working digestion mixture, a stock digestion solution is first prepared and can be stored at −20°C for up to 6 months.

**Table:** Stock digestion mixture (Ex. 40 mL)

Reagent	Final concentration	Amount
RPMI medium	N/A	40 mL
Collagenase-Type I	10 mg/mL	400 mg
Dispase II	10 mg/mL	400 mg
**Total**	**N/A**	**40 mL**

***Note:*** Make sure that collagenase-type I and Dispase II are thoroughly dissolved and mixed before proceeding. Once dissolved and mixed, sterile filter stock digestion mixture using a 50-mL Millipore Steriflip-GP Sterile Centrifuge Tube Top Filter Unit (0.22-μM, Millipore, SCGP00525). Aliquot 1 mL into Sarstedt 1.5-mL Screw Cap Micro Tube (Fisher Scientific, 50-809-238) and freeze at −20°C for long-term storage.

**Table:** Working digestion mixture (Ex. 10 mL)

Reagent	Final concentration	Amount
RPMI medium	N/A	9 mL
Stock digestion mixture (10 mg/mL)	1 mg/mL	1 mL
DNase I	1 mg/mL	10 mg
**Total**	**N/A**	**10 mL**

***Note:*** When primary samples need to be digested, the working digestion mixture is freshly prepared and used immediately.

## Step-by-step method details

### Human OC single-cell processing

**Timing: 2–4 h**

These steps outline the methods to prepare single cells from human OC ascites or pleural fluid samples (Figure 1).

1. Resuscitate cryopreserved human OC ascites or pleural fluid samples. Troubleshooting 1.
  a. Obtain cryopreserved sample from −80°C or liquid nitrogen and quickly thaw samples in the 37°C water bath.
  b. Wash out sample with RPMI media and pipette into 15-mL conical tube.
  c. Centrifuge at 500 x g for 5 min.
  d. Resuspend in RPMI media without supplements and aliquot small volume to determine cell count, viability, and aggregated state of tumor cells.
    ***Note:*** Tumor cells often aggregate together in ascites or pleural fluid and as such, this step is to determine whether aggregates are present and if digestion is required.
  e. Once counted, add additional RPMI media to wash out sample further and centrifuge at 500 x g for 5 min.
  f. If digestion is necessary, aspirate and proceed to step 2a. However, if digestion is not necessary, proceed to step 3d.
2. Digest aggregated tumor cells in human OC ascites or pleural fluid, as determined in 1d. Troubleshooting 2*.*
  a. If digestion has been determined as necessary, resuspend cell pellet in working digestion mixture.
  b. Incubate for 60 min at 37°C while gently rotating.
    i. Aliquots of sample can be taken at intervals to determine whether additional digestion time is necessary.

**CRITICAL:** Proper dissociation of aggregates is crucial to accurately represent the tumor population in the sample, therefore additional time may be necessary for increased dissociation. However, extended periods of time spent digesting can reduce viability. Therefore, these two points must be carefully weighed.

3. Preparing digested human OC ascites or pleural fluid for downstream analyses. Troubleshooting 3*.*
  a. Following digestion, centrifuge the sample at 500 x g for 5 min.
  b. Aspirate supernatant and resuspend cell pellet in RPMI media to determine cell count and viability.
  c. Wash with additional RPMI media and centrifuge at 500 x g for 5 min.
  d. Aspirate supernatant and prepare sample for downstream analyzes.

### Human OC tumor and TME profiling

**Timing: 2–4 h**

These steps outline the methods for profiling primary OC tumor cells and their TME using flow cytometry.

**Figure 4:**
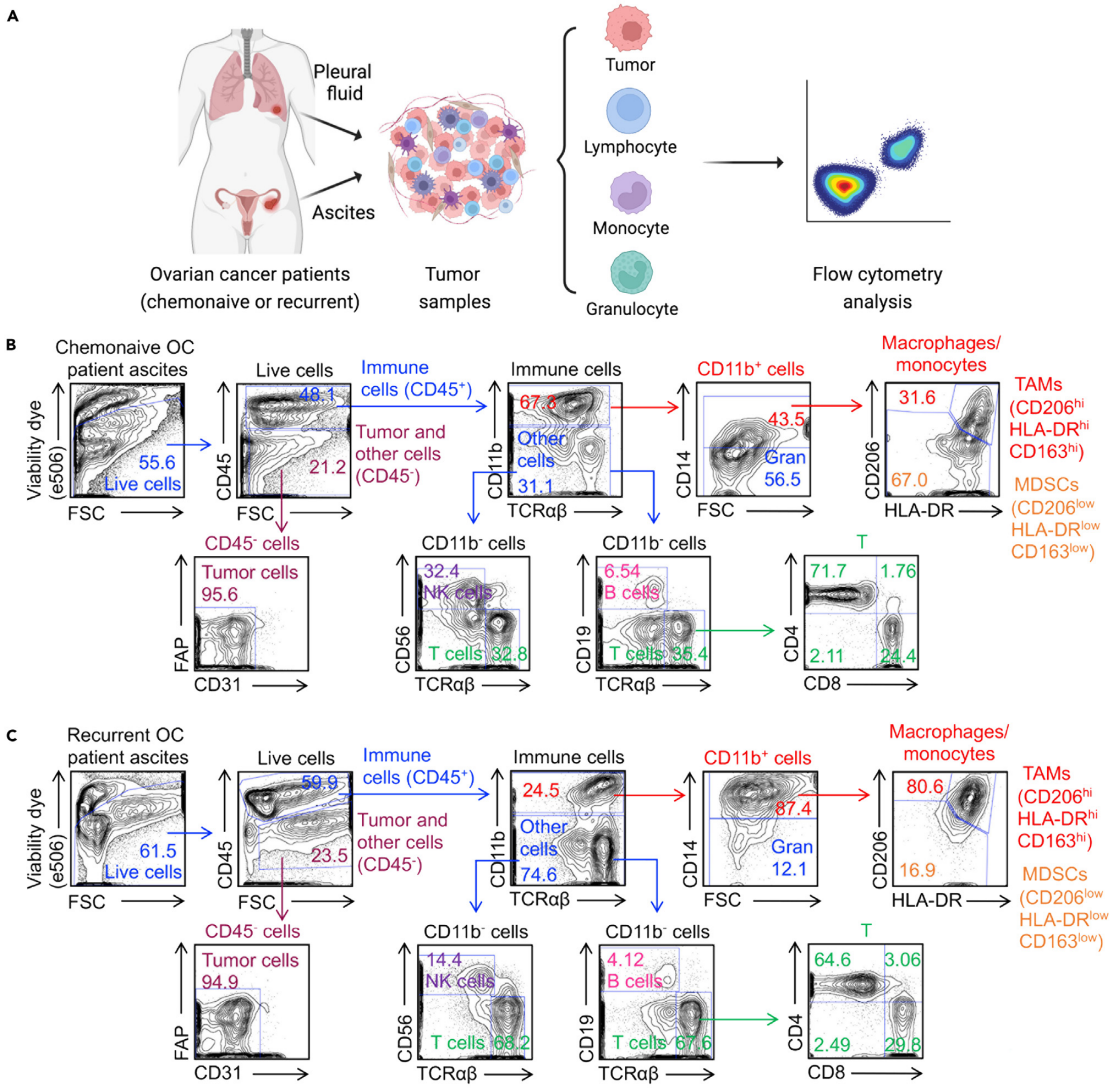
Profiling primary OC tumor cells and their tumor microenvironment (TME) (A) Schematics showing the experimental design of collecting and studying tumor cells and their TME in primary OC patient samples. (B and C) FACS analyses of the chemonaive (B) and recurrent (C) OC patient samples. Tumor cells are identified as CD45^-^ cells; immune cells are identified as CD45^+^ cells; tumor-associated macrophages (TAMs) are identified as CD45^+^CD11b^+^CD14^+^CD206^hi^HLA-DR^hi^CD163^hi^ cells; myeloid-derived suppressor cells (MDSCs) are identified as CD45^+^CD11b^+^CD14^+^CD206^low^HLA-DR^low^CD163^low^ cells; B cells are identified as CD45^+^ CD11b^-^CD19^+^ or CD45^+^ CD11b^-^CD20^+^ cells; NK cells are identified as CD45^+^CD11b^-^CD56^+^ cells; T cells are identified as CD45^+^ CD11b^-^CD3^+^ cells. Representative of >20 experiments.

4. OC tumor and TME cell preparation.
  a. Thaw cryopreserved human OC cells quickly in a 37°C water bath.
  b. Transfer the cells into a 15-mL conical tube containing 10 mL pre-warmed C10 medium.
  c. Centrifuge cells at 300 × g for 5 min. Aspirate the supernatant completely.
  d. Resuspend in C10 medium. Transfer cells into a 5-mL FACS tube.
  e. Centrifuge at 300 × g for 5 min and aspirate supernatant.
5. OC tumor and TME cell staining.
  a. Resuspend the cells in 100 μL 1 x PBS buffer.
  b. Stain cells with the following:
    i. Fixable Viability Dye eFluor506 (Affymetrix eBioscience, cat. no. 65-0866-14, 1:500 dilution).
    ii. Human Fc Receptor Blocking Solution (TrueStain FcX) (BioLegend, cat. no. 422302, 1:100 dilution).
      ***Note:*** All antibodies used for cell staining are diluted in 1x PBS buffer.
  c. Gently vortex and incubate on ice for 20–30 min in the dark.
  d. Wash cells with 1–2 mL 1 x PBS buffer, centrifuge at 300 × g for 5 min, and aspirate supernatant. Resuspend the cells in 100 μL 1 x PBS buffer.
  e. Stain cells with the following antibodies (Figures 4B and 4C; Tables 1 and 2):
    i. PerCP-conjugated anti-CD45 (BioLegend, cat. no. 304026, 1:500 dilution).
    ii. FITC-conjugated anti-CD11b (BioLegend, cat. no. 301330, 1:500 dilution).
    iii. APC-conjugated anti-CD14 (BioLegend, cat. no. 325608, 1:100 dilution).
    iv. APC-conjugated anti-CD206 (BioLegend, cat. no. 321110, 1:100 dilution).
    v. APC-Cy7-conjugated anti-HLA-DR (BioLegend, cat. no. 307618, 1:250 dilution).
    vi. APC-Cy7-conjugated anti-CD163 (BioLegend, cat. no. 333621, 1:50 dilution).
    vii. APC-conjugated anti-CD19 (BioLegend, cat. no. 363005, 1:100 dilution).
    viii. FITC-conjugated anti-CD56 (BioLegend, cat. no. 362545, 1:10 dilution).
    ix. Pacific Blue-conjugated anti-CD3 (BioLegend, cat. no. 300329, 1:500 dilution).
      ***Note:*** This step involves identifying human OC tumor cells, TAMs, MDSCs, B cells, NK cells, and T cells (Figures 4B and 4C; Tables 1 and 2). Additional antibodies or fluorescent markers can be incorporated depending on the specific experimental design.
      ***Note:***Table 1 lists the markers of OC tumor cells, including CAR targets, NK ligands, TCR targets, and CSC markers. Additional markers may also be considered for testing.

**Table 1:** Tumor antigens detected on primary OC tumor cells

Antigen category	Antigen	Description of the antigen roles in OC	Included in the research article^1^?
CAR antigens	Mesothelin (MSLN)	A tumor-associated protein expressed on OC cells, making it a potential CAR target for immunotherapy and therapeutic interventions	Yes
	MUC16 (CA125)	A glycoprotein that is overexpressed on the surface of OC cells and serves as a biomarker for the diagnosis and monitoring of OC, as well as a potential target for targeted therapies and immunotherapy strategies	Yes
	HER2	A receptor tyrosine kinase that is frequently overexpressed in certain subtypes of OC, serving as a significant target for targeted therapies and immunotherapeutic approaches, including CAR T cell therapies	No
	FRα (Folate receptor alpha)	A cell surface glycoprotein that is commonly overexpressed on OC cells and other epithelial tumors, serving as a critical target for targeted therapies	No
	EpCAM	A cell surface protein that is frequently overexpressed on epithelial tumor cells, making it a potential target for immunotherapeutic strategies	No
	TROP2	A cell surface glycoprotein that is often overexpressed on OC cells, making it an attractive target for therapeutic strategies, including CAR T cell therapies and antibody-drug conjugates	No
ESO TCR antigen	NY-ESO-1	A cancer/testis antigen that is expressed in various tumors, including OC, and is primarily found in germ cells; it is of particular interest for immunotherapy due to its restricted expression in normal tissues and its potential to elicit a strong immune response, making it a target for cancer vaccines and adoptive T cell therapies	Yes
NK ligands	ULBP	A family of ligands, including ULBP1, 2, and 3, which are stress-inducible proteins found on the surface of certain tumor cells, including OC; these ligands interact with the NKG2D on NK cells and cytotoxic T lymphocytes, facilitating immune recognition and response against cancer cells	Yes
	MICA/B	Stress-induced ligands that are expressed on the surface of various tumor cells, including OC cells; They interact with the NKG2D receptor on NK cells and certain T cells, playing a crucial role in immune surveillance by promoting the recognition and elimination of tumor cells	Yes
	CD112 (Nectin-2)	An immunoglobulin-like molecule expressed on OC cells that interacts with DNAM-1 on immune cells, potentially contributing to tumor immune evasion and serving as a target for immunotherapy	Yes
	CD155 (PVR)	An immunoglobulin-like protein expressed on the surface of various tumor cells, including OC cells, that interacts with DNAM-1 on NK cells and T cells, thus playing a role in immune evasion and tumor progression, and presenting an opportunity for targeted immunotherapy strategies	Yes
Cancer stem cell (CSC) markers	SOX2	Transcription factors that are crucial for maintaining pluripotency and self-renewal in stem cells, and its expression has been associated with OC cells, where it may contribute to tumorigenesis, cell differentiation, and resistance to therapy	Yes
	OCT3/4		Yes
	Nanog		Yes
	CD24	Cell surface markers frequently associated with OC and tumor stem cells; CD24 is often linked to tumor progression and metastasis, CD44 plays a role in cell adhesion and migration, CD117 (c-KIT) is a receptor tyrosine kinase involved in cell signaling and survival, and CD133 is considered a marker of cancer stem cells, indicating the presence of a stem-like phenotype that contributes to tumor initiation and resistance to therapy	Yes
	CD44		Yes
	CD117		Yes
	CD133		Yes

**Table 2:** Markers are used to identify cells within OC tumor cells and the TME

Cell type	Flow markers
Tumor cell	CD45^-^FAP^-^CD31^-^
Immune cell	CD45^+^
T cell	CD45^+^CD3^+^(TCR^+^)
Helper T cell	CD45^+^CD3^+^(TCR^+^)CD4^+^
Cytotoxic T cell	CD45^+^CD3^+^(TCR^+^)CD8^+^
B cell	CD45^+^CD3^-^CD19^+^ or CD45^+^CD3^-^CD20^+^
NK cell	CD45^+^CD3^-^CD56^+^
Tumor-associated macrophage (TAM)	CD45^+^CD3^-^CD11b^+^CD14^+^HLA-DR^hi^CD206^hi^CD163^hi^
Monocytic myeloid-derived suppressor cell (MDSC)	CD45^+^CD3^-^CD11b^+^CD14^+^HLA-DR^low^CD206^low^CD163^low^
Granulocyte	CD45^+^CD3^-^CD11b^+^CD14^-^CD15^+^
  f. Gently vortex and incubate on ice for 20–30 min in the dark.
  g. Wash cells with 1–2 mL 1 x PBS buffer, centrifuge at 300 × g for 5 min, and aspirate supernatant.
  h. Resuspend the cells in 200 μL 1 x PBS buffer.
6. Flow cytometry analysis.
  a. Exclude dead cells (e506^+^ cells).
  b. Gate and quantify the OC tumor cells, TAMs, MDSCs, B cells, NK cells, and T cells according to the gating strategy from Figures 4B and 4C.

### *In vitro* OC tumor cell killing assays

**Timing: 2 days**

These steps outline the methods for evaluating various cell-based therapies targeting primary OC tumor cells using *in vitro* killing assays.

**Figure 5:**
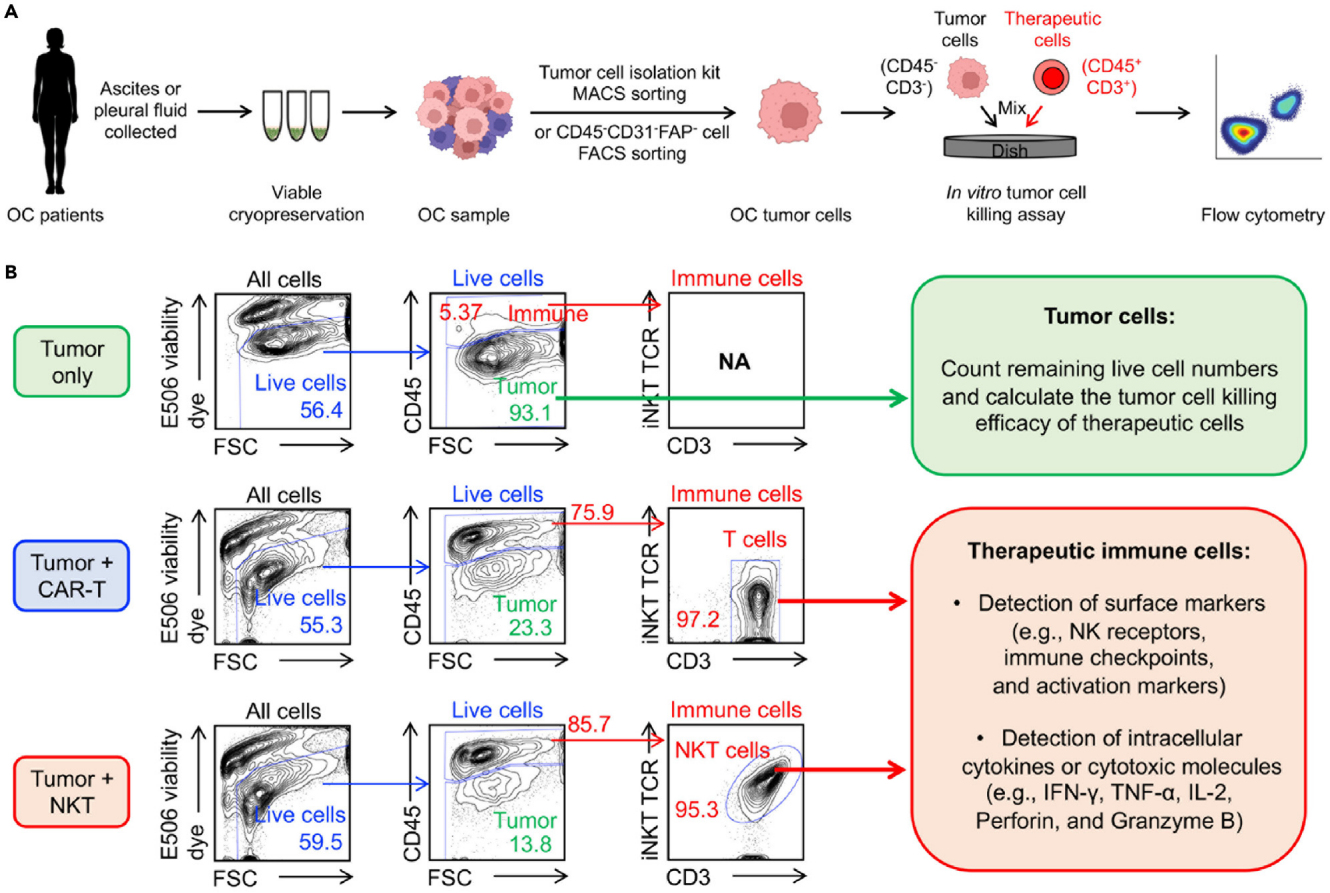
Evaluate cell-based immunotherapy on primary OC tumor cells (A) Experimental design to evaluate the anti-tumor efficacy of therapeutic cells (e.g., CAR-T and NKT cells) on primary OC tumor cells. (B) FACS analyses of OC tumor cells and therapeutic cells following 24 h of co-culture. Representative of >20 experiments.

***Note:*** Two methods, including Magnetic-activated cell sorting (MACS) and Fluorescence-activated cell sorting (FACS), are both available for tumor cell isolation (Figure 5A).

7. Tumor cell isolation using Tumor Cell Isolation Kit (Miltenyi Biotec, cat. no. 130-108-339) and MACS sorting. The details of these steps can be found in the manufacturer’s instructions. Troubleshooting 4*.*
  a. Thaw cryopreserved human OC cells quickly in a 37°C water bath.
  b. Transfer the cells into a 15-mL conical tube containing 10 mL pre-warmed C10 medium.
  c. Determine the cell number using a hemacytometer.
  d. Centrifuge cells at 300 × g for 10 min. Aspirate the supernatant completely.
  e. Resuspend up to 10^7^ total cells in MACS buffer.
  f. Add 20 μL Non-Tumor Cell Depletion Cocktail A and 20 μL Non-Tumor Cell Depletion Cocktail B.
  g. Mix well and incubate the cell suspension at 2°C–8°C for 15 min.
  h. Adjust the volume to 500 μL with MACS buffer.
    **CRITICAL:** Keep cells on ice during the labeling step to preserve viability.
  i. Place an LS column into the magnetic field of a MACS Separator.
  j. Rinse the column with 3 mL MACS buffer.
  k. Apply the labeled cell suspension to the column. Collect the flow-through (unlabeled tumor cells).
  l. Wash the column twice with 1 mL buffer. Combine flow-through fractions.
8. FACS sorting using surface marker staining.
  a. Thaw cryopreserved tumor cells as described above.
  b. Transfer cells into a 5-mL FACS tube. Centrifuge at 300 × g for 5 min and aspirate supernatant.
  c. Resuspend the cells in 100 μL 1 x PBS buffer.
  d. Add the following antibodies:
    i. Fixable Viability Dye eFluor506 (Affymetrix eBioscience, cat. no. 65-0866-14, 1:500 dilution).
    ii. Human Fc Receptor Blocking Solution (TrueStain FcX) (BioLegend, cat. no. 422302, 1:100 dilution).
  e. Gently vortex and incubate on ice for 20–30 min in the dark.
  f. Wash cells with 1–2 mL 1 x PBS buffer, centrifuge at 300 × g for 5 min, and aspirate supernatant. Resuspend the cells in 100 μL 1 x PBS buffer.
  g. Add the following antibodies:
    i. PerCP-conjugated anti-CD45 (BioLegend, cat. no. 304026, 1:500 dilution).
    ii. PE-Cy7-conjugated anti-CD31 (BioLegend, cat. no. 303117, 1:100 dilution).
    iii. Fluorochrome-unconjugated anti-FAP (R&D Systems, cat. no. MAB3715-SP, 1:100 dilution).
      ***Note:*** This step involves isolating human immune cells (CD45^+^ cells), fibroblast cells (FAP^+^ cells), and endothelial cells (CD31^+^ cells). Additional antibodies or fluorescent markers can be incorporated depending on the specific experimental design.
  h. Gently vortex and incubate on ice for 20–30 min in the dark.
  i. Wash cells with 1–2 mL 1 x PBS buffer, centrifuge at 300 × g for 5 min, and aspirate supernatant. Resuspend the cells in 100 μL 1 x PBS buffer.
  j. Add the FITC-conjugated goat anti-mouse IgG (BioLegend, cat. no. 405305, 1:500 dilution).
  k. Gently vortex and incubate on ice for 20–30 min in the dark.
  l. Wash cells with 1–2 mL 1 x PBS buffer, centrifuge at 300 × g for 5 min, and aspirate supernatant.
  m. Resuspend cells in 500 μL 1 x PBS buffer for sorting.
  n. Set up the FACS sorter to exclude dead (e506^+^ cells) and non-tumor cells (CD45^+^, CD31^+^, and FAP^+^ cells).
  o. Collect e506^−^CD45^−^CD31^−^FAP^−^ OC tumor cells into sterile tubes containing 500 μL 1 x PBS buffer.
    **CRITICAL:** Use appropriate gating controls (unstained and single stains) to ensure accurate gating.
9. OC tumor cell preparation.
  a. Thaw or freshly isolate tumor cells. Resuspend in C10 medium.
  b. Count tumor cells and adjust the concentration to 1 × 10^6^ cells/mL.
10. Therapeutic cell preparation.
  a. Prepare therapeutic cells (e.g., MCAR-T, ESO-T, or NKT cells) in C10 medium.
  b. Count therapeutic cells and adjust the therapeutic cell concentration to 1 × 10^6^ cells/mL.
11. Co-culture setup.
  Step instruction: Co-culture tumor and therapeutic cells in a round-bottom 96-well plate.
  a. Set up the following effector:tumor (E:T) ratios in triplicate: MCAR-T cells: 1:1 ratio (1 × 10^4^ tumor cells + 1 × 10^4^ MCAR-T cells); ESO-T cells: 2:1 ratio (1 × 10^4^ tumor cells + 2 × 10^4^ ESO-T cells); NKT cells: 2:1 ratio (1 × 10^4^ tumor cells + 2 × 10^4^ NKT cells).
    ***Note:*** The E:T ratios and cell numbers could be adjusted based on the experimental designs.
    ***Note:*** Therapeutic cells from multiple donors should be tested to ensure the reliability of the assays.
  b. Add C10 medium to adjust the final volume to 200 μL per well.
  c. Incubate the 96-well plate at 37°C, 5% CO_2_ for 24 h.
12. Quantification of live OC tumor cells using flow cytometry after the 24-h co-culture.
  a. Cell staining.
    i. Centrifuge the 96-well plate at 300 × g for 5 min at 4°C.
    ii. Aspirate the supernatant.
    iii. Stain cells with Fixable Viability Dye eFluor506 (Affymetrix eBioscience, cat. no. 65-0866-14, 1:500 dilution) and PerCP-conjugated anti-CD45 (BioLegend, cat. no. 304026, 1:500 dilution).
      ***Note:*** Various fluorescence-labeled anti-human CD45 antibodies can also be used.
    iv. Incubate samples on ice for 20 min in the dark.
    v. Wash with 1 x PBS buffer, centrifuge, and resuspend in 50 μL 1 x PBS buffer.
  b. Flow cytometry analysis.
    i. Exclude dead cells (e506^+^ cells).
    ii. Gate and quantify the live OC tumor cells as CD45^-^ cells and live therapeutic cells as CD45^+^ cells (Figure 5B).

### *In vitro* OC TAM/MDSC killing assays

**Timing: 2 days**

These steps outline the methods for evaluating human NKT cell-based therapy targeting primary OC TME cells using *in vitro* killing assays.

**Figure 6:**
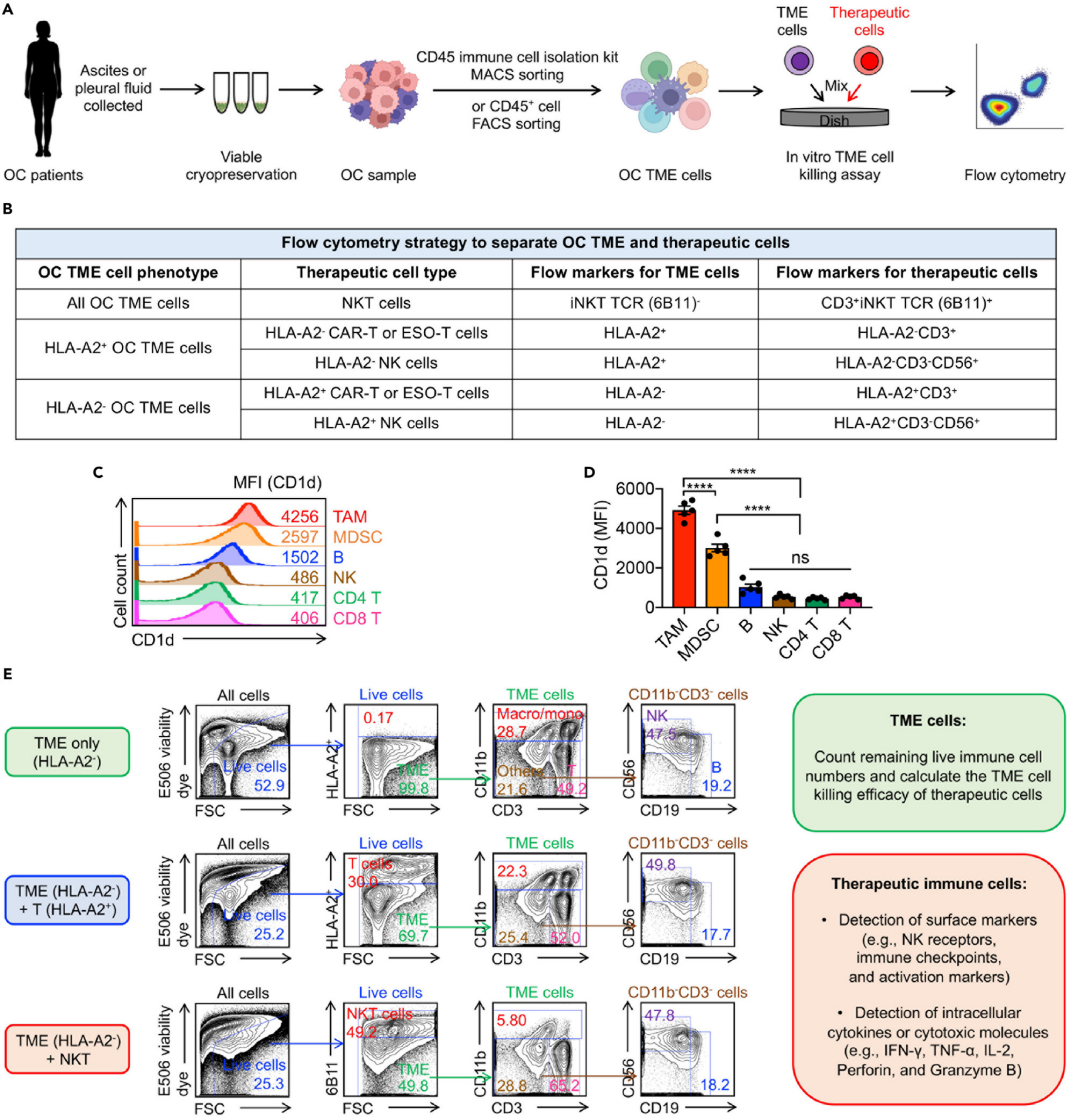
Evaluate cell-based immunotherapy on primary OC TME (A) Experimental design to evaluate the anti-tumor efficacy of therapeutic cells (e.g., T and NKT cells) on primary OC TME immune cells. (B) Table summarizing the flow cytometry strategy for gating OC TME cells and therapeutic cells. (C) FACS analyses of CD1d expression on the indicated OC TME immune cells. (D) Quantification of (C) (*n* = 5). (E) FACS analyses of OC TME immune cells and therapeutic cells following 24 h of co-culture. Representative of >20 experiments. Data are presented as the mean ± SEM. ns, not significant, ∗∗∗∗*p* < 0.0001, by one-way ANOVA (D).

***Note:*** Two methods, including MACS and FACS, are both available for immune cell isolation (Figure 6A).

13. CD45^+^ immune cell isolation using Human CD45 MicroBeads Kit (Miltenyi Biotec, cat. no. 130-045-801) and MACS sorting. The details of these steps can be found in the manufacturer’s instructions.
  a. Thaw cryopreserved human OC cells quickly in a 37°C water bath.
  b. Transfer the cells into a 15-mL conical tube containing 10 mL pre-warmed C10 medium.
  c. Determine the cell number using a hemacytometer.
  d. Centrifuge cells at 300 × g for 10 min. Aspirate the supernatant completely.
  e. Resuspend cells in 80 μL MACS buffer per 10^7^ total cells.
  f. Add 20 μL CD45 Microbeads per 10^7^ total cells.
  g. Mix well and incubate the cell suspension at 2°C–8°C for 15 min.
  h. Wash cells by adding 1–2 mL MACS buffer per 10^7^ total cells.
  i. Centrifuge at 300 × g for 10 min. Aspirate the supernatant completely.
  j. Resuspend up to 10^8^ cells in 50 μL MACS buffer.
    **CRITICAL:** Keep cells on ice during the labeling step to preserve viability.
  k. Place an MS or LS column (choose according to the total cell number and CD45^+^ cell number) into the magnetic field of a MACS Separator.
  l. Rinse the column with 500 μL (MS) or 3 mL (LS) MACS buffer.
  m. Apply the labeled cell suspension to the column.
  n. Wash the column for three times with 500 μL (MS) or 3 mL (LS) MACS buffer.
  o. Remove column from the separator and place it on a 15-mL conical tube.
  p. Pipette 1 mL (MS) or 5 mL (LS) MACS buffer onto the column. Immediately flush out the magnetically labeled cells by firmly pushing the plunger into the column.
14. FACS sorting using surface marker staining.
  a. Thaw cryopreserved OC cells as described above.
  b. Transfer cells into a 5-mL FACS tube. Centrifuge at 300 × g for 5 min and aspirate supernatant.
  c. Resuspend the cells in 100 μL 1 x PBS buffer.
  d. Add the following antibodies:
    i. Fixable Viability Dye eFluor506 (Affymetrix eBioscience, cat. no. 65-0866-14, 1:500 dilution).
    ii. Human Fc Receptor Blocking Solution (TrueStain FcX) (BioLegend, cat. no. 422302, 1:100 dilution).
  e. Gently vortex and incubate on ice for 20–30 min in the dark.
  f. Wash cells with 1–2 mL 1 x PBS buffer, centrifuge at 300 × g for 5 min, and aspirate supernatant. Resuspend the cells in 100 μL 1 x PBS buffer.
  g. Add the following antibodies: PerCP-conjugated anti-CD45 (BioLegend, cat. no. 304026, 1:500 dilution).
    ***Note:*** This step involves isolating human immune cells (CD45^+^ cells). Additional antibodies or fluorescent markers can be incorporated depending on the specific experimental design.
  h. Gently vortex and incubate on ice for 20–30 min in the dark.
  i. Wash cells with 1–2 mL 1 x PBS buffer, centrifuge at 300 × g for 5 min, and aspirate supernatant.
  j. Resuspend cells in 500 μL 1 x PBS buffer for sorting.
  k. Set up the FACS sorter to exclude dead (e506^+^ cells) and non-immune cells (CD45^−^ cells).
  l. Collect e506^−^CD45^+^ OC TME cells into sterile tubes containing 500 μL 1 x PBS buffer.
    **CRITICAL:** Use appropriate gating controls (unstained and single stains) to ensure accurate gating.
15. *In vitro* OC TAM/MDSC killing assay.
  a. OC TME cell preparation.
    i. Thaw or freshly isolate OC TME cells. Resuspend in C10 medium.
    ii. Count TME cells and adjust the concentration to 1 × 10^6^ cells/mL.
  b. Therapeutic cell preparation.
    i. Prepare therapeutic cells (e.g., NKT or conventional T cells) in C10 medium.
    ii. Count therapeutic cells and adjust the therapeutic cell concentration to 1 × 10^6^ cells/mL.
  c. Co-culture setup.
    Step instruction: Co-culture TME and therapeutic cells in a round-bottom 96-well plate.
    i. Set up the following therapeutic cell:TME cell ratios in triplicate: NKT cells: 1:1 ratio (1 × 10^5^ TME cells + 1 × 10^5^ NKT cells); T cells: 1:1 ratio (1 × 10^5^ TME cells + 1 × 10^5^ T cells).
      ***Note:*** The therapeutic cell: TME cell ratios and cell numbers could be adjusted based on the experimental designs.
      ***Note:*** Therapeutic cells from multiple donors should be tested to ensure the reliability of the assays.
    ii. Add C10 medium to adjust the final volume to 200 μL per well.
    iii. Incubate the 96-well plate at 37°C, 5% CO_2_ for 24 h.
16. Quantification of live TME cells using flow cytometry after the 24-h co-culture.
  a. Centrifuge the 96-well plate at 300 × g for 5 min at 4°C. Aspirate the supernatant.
  b. Stain cells with the following:
    i. Fixable Viability Dye eFluor506 (Affymetrix eBioscience, cat. no. 65-0866-14, 1:500 dilution).
    ii. APC-Cy7-conjugated anti-HLA-A2 (BioLegend, cat. no. 343310, 1:500 dilution).
    iii. Pacific Blue-conjugated anti-CD3 (BioLegend, cat. no. 300329, 1:500 dilution).
    iv. PE-conjugated anti-6B11 (BioLegend, cat. no.552825, 1:10 dilution).
    v. APC-conjugated anti-CD19 (BioLegend, cat. no. 363005, 1:100 dilution).
    vi. FITC-conjugated anti-CD11b (BioLegend, cat. no. 301330, 1:500 dilution).
    vii. APC-conjugated anti-CD19 (BioLegend, cat. no. 363005, 1:100 dilution).
      ***Note:*** Various fluorescence-labeled antibodies can also be used. Additional antibodies or fluorescent markers can be incorporated depending on the specific experimental design.
  c. Incubate samples on ice for 20 min in the dark.
  d. Wash with 1 x PBS buffer, centrifuge, and resuspend in 50 μL 1 x PBS buffer.
  e. Flow cytometry analysis.
    i. Exclude dead cells (e506^+^ cells).
    ii. Gate and quantify the live TAM and MDSC cells following the gating strategy from Figure 6B and 6E, and Table 2.
      ***Note:*** In this protocol, the cytotoxic activity against various OC TME cell types is assessed, with a particular focus on targeting TAMs and MDSCs. These cells are known for their immunosuppressive properties, which contribute to tumor progression and impede therapeutic efficacy.^16^ As such, this experiment is designated as the *in vitro* OC TAM/MDSC killing assay. Cytotoxicity against other cell types, including T cells, B cells, and NK cells, will also be evaluated as controls.

## Expected outcomes

This protocol enables the generation and evaluation of antigen-specific T cells, including healthy donor PBMC-derived mesothelin-targeting MCAR-T cells and NY-ESO-1-targeting ESO-T cells, through gene engineering and *in vitro* tumor cell killing assays (Figures 2A and 2E). Lentiviral transduction of PBMC-derived T cells, combined with anti-CD3/CD28 stimulation, efficiently produces MCAR-T or ESO-T cells with high CAR or ESO TCR expression, respectively (Figures 2B and 2F). MCAR-T cells are anticipated to demonstrate robust cytotoxicity against mesothelin-expressing tumor cell lines, such as OVCAR3-FG and OVCAR8-FG, while sparing mesothelin-negative tumor cells like SKOV3-FG (Figures 2C and 2D). Similarly, ESO-T cells are expected to exhibit potent cytotoxicity against NY-ESO-1-positive, HLA-A2-restricted tumor cells, including OVCAR3-A2-ESO-FG and A375-A2-ESO-FG, with minimal activity against NY-ESO-1-negative tumor cells (Figures 2G and 2H).

This protocol also emphasizes the development and characterization of human allogeneic NKT cells generated using HSPC gene engineering and a clinically guided culture method (Figure 3A).^11^^,^^17^^,^^18^^,^^19^^,^^20^ The generated NKT cells express >98% iNKT TCR, as confirmed by flow cytometry (Figure 3B), validating their identity and purity. These cells exhibit enhanced expression of NK receptors, including CD56, NKG2D, DNAM-1, and NKp44, underscoring their heightened cytotoxic potential against a range of tumor types (Figures 3C and 3D). Functional assays reveal superior tumor cell killing efficacy of NKT cells compared to PBMC-derived conventional T cells, targeting tumor cell lines including OVCAR3-FG, OVCAR8-FG, SKOV3-FG, A375-FG, PC3-FG, Raji-FG, and K562-FG. (Figures 3E and 3F) Blocking studies further confirm the critical roles of NKG2D and DNAM-1 in mediating NKT cytotoxicity, as inhibition of these NK receptors significantly reduces their tumor cell killing efficiency against OVCAR3-FG cells (Figure 3G).

In addition, this protocol enables comprehensive immune profiling of tumor cells and their TME from primary OC patients, encompassing chemonaive and recurrent diseases (Figure 4A). Pleural fluid and ascites OC samples are processed to identify tumor cells and their TME immune cells (e.g., monocytes, B cells, T cells, NK cells, TAMs, MDSCs, and granulocytes) using flow cytometry (Figures 4B and 4C). Notably, recurrent OC ascites exhibit an increased proportion of TAMs and MDSCs, highlighting their role in fostering an immunosuppressive TME (Figures 4B and 4C).

We also evaluate the OC tumor cells and their TME targeting using a series of *in vitro* killing assays. OC tumor cells (identified as CD45^-^CD31^-^FAP^-^ cells) are isolated using a human tumor cell isolation kit or FACS sorting, and then co-cultured with therapeutic immune cells, such as CAR-T or NKT cells (Figure 5A). Flow cytometry is used to evaluate tumor cell killing capacity of therapeutic cells and their immune phenotypes. NKT cells exhibit superior tumor cell killing efficacy compared to conventional CAR-T cells, particularly in targeting immunosuppressive tumor microenvironments (Figure 5B).^1^^,^^21^

In order to evaluate the OC TME targeting by the therapeutic cells, the TME immune cells could be isolated using the CD45 immune cell isolation kit or FACS sorting (Figure 6A). Flow cytometry distinguishes immune cell subsets within the OC TME, including TAMs, MDSCs, T cells, NK cells, and B cells, based on surface marker expression (Figure 6B). Notably, high CD1d expression on TAMs and MDSCs highlights these subsets as key targets for NKT cells via CD1d/TCR recognition (Figures 6C and 6D). In the *in vitro* co-culture assays, NKT cells demonstrate enhanced cytotoxic activity against TAMs and MDSCs compared to conventional CAR-T cells, indicating their potent anti-OC capacity (Figure 6E).

## Quantification and statistical analysis

A Prism 8 software (GraphPad) was utilized for all statistical analysis. Pairwise comparisons were performed with a 2-tailed Student’s *t* test. Multiple comparisons were performed with an ordinary 1-way ANOVA, followed by Tukey’s multiple comparisons test. Unless otherwise indicated, data are presented as the meanGSEM. In all figures and figure legends, ‘‘n’’ represents the number of biological replicates in which the experiment was performed. A *p* value of less than 0.05 was considered significant. ns, not significant; ∗*p* < 0.05; ∗∗*p* < 0.01; ∗∗∗*p* < 0.001; ∗∗∗∗*p* < 0.0001.

## Limitations

This protocol utilizes primary OC patient samples, specifically focusing on live tumor cells and immune cells within the OC TME. Consequently, it necessitates high cell viability from these primary samples, as the presence of dead cells can significantly compromise the integrity of the results. Close collaboration between clinical and research teams is essential to ensure the proper handling and storage of these primary samples. Nonetheless, patient-to-patient variability may introduce inconsistencies between OC samples.

The protocol incorporates flow cytometry for the profiling of surface antigens and transcription factors on OC tumor cells; however, it is important to note that additional markers may not be included in this analysis. Employing unbiased profiling techniques, such as RNA sequencing, could yield comprehensive insights into the status and phenotype of the tumor cells. Moreover, this protocol currently evaluates *in vitro* cytotoxicity using three therapeutic cell types: CAR-T, ESO-T, and NKT cells. Additional therapeutic cell types, such as NK cells, CAR-NK cells, and CAR macrophages, should also be investigated. The results of the killing assays are primarily derived from flow cytometry; however, other methodologies, such as lactate dehydrogenase (LDH) release assay and Calcein-AM assay, are available to quantify the killing capacity of the various therapeutic cells.

## Troubleshooting

### Problem 1

Low viability upon resuscitating cryopreserved human OC ascites and pleural fluid samples. Related to “human OC single-cell processing” step (1).

### Potential solution

If the viability of cryopreserved samples has dropped drastically between the time of freezing and resuscitation, this may be a result of inadequate freezing down of the sample. To ensure optimal viability upon resuscitation, be sure to freeze sample down quickly, keep all reagents and materials on ice, and keep concentration of live cells per vial below 1 × 10^7^ cells/mL.

### Problem 2

Poor dissociation of tumor aggregates from human OC ascites or pleural fluid samples. Related to “human OC single-cell processing” step (2).

### Potential solution

This may be caused by insufficient digestion time of the sample. We recommend digesting for additional time, 15–30 min, and checking to see if tumor cells are adequately separated.

### Problem 3

Low viability of human OC ascites or pleural fluid sample following digestion step. Related to “human OC single-cell processing” step (3).

### Potential solution

A low sample viability following digestion may be due to an over digestion of the sample. Reducing the digestion time may enhance sample viability. For fragile samples, periodically check the viability of the sample to reduce cell death.

### Problem 4

Low cell number recovery following tumor cell isolation via column-based methods. Related to “in vitro OC tumor cell killing assays” step (7).

### Potential solution

Following tumor cell isolation, if the tumor cell recovery is low, if not the biological nature of the sample, may be due to an increased aggregated presence in the sample. Tumor aggregates can get stuck in column-based isolation methods of tumor cells. Following elution of sample, into another tube, overlay additional elution buffer and expunge sample contained with the column. Determine whether aggregates are present and if additional cells are needed, consider re-digesting these aggregates again following the digestion protocol.

## Resource availability

### Lead contact

Further information and requests for resources and reagents should be directed to and will be fulfilled by the lead contact, Lili Yang (liliyang@ucla.edu).

### Technical contact

Technical questions on executing this protocol should be directed to and will be answered by the technical contact, Yan-Ruide Li (charlie.li@ucla.edu).

### Materials availability

Human tumor cell lines generated in this study will be made available upon reasonable request.

### Data and code availability

This study did not generate new datasets or code.

## Acknowledgments

We thank the University of California, Los Angeles (UCLA) CFAR Virology Core for providing human cells and the UCLA BSCRC Flow Cytometry Core Facility for cell sorting support. We would like to acknowledge the National Institutes of Health (NIH) Division of Cancer Treatment and Diagnosis (DCTD) Tumor Repository for providing the OVCAR8 cell line. This work was supported by a Partnering Opportunity for Discovery Stage Award from the California Institute for Regenerative Medicine (DISC2-13505 to L.Y.), a UCLA BSCRC Stem Cell Research Innovation Award (to L.Y. and S.M.), and an Ablon Scholars Award (to L.Y.). L.Y. is an investigator of the Parker Institute for Cancer Immunotherapy (PICI) at UCLA. Y.-R.L. is a postdoctoral fellow supported by a UCLA MIMG M. John Pickett Post-Doctoral Fellow Award, a CIRM-BSCRC Postdoctoral Fellowship, a UCLA Sydney Finegold Postdoctoral Award, and a UCLA Chancellor’s Award for Postdoctoral Research. C.J.O. is a predoctoral fellow supported by a CIRM-BSCRC Predoctoral Fellowship. Some figures were created with BioRender (biorender.com).

## Author contributions

Y.-R.L., C.J.O., S.M., and L.Y. designed the experiments, analyzed the data, and wrote the manuscript. S.M. and L.Y. conceived and oversaw the study, with assistance from Y.-R.L. and C.J.O. Y.-R.L. and C.J.O. performed experiments and wrote the manuscript, with assistance from Z.L., Y.Z., Y.C., G.D., and L.R.

## Declaration of interests

Y.-R.L. and L.Y. are inventors on patents relating to this manuscript. L.Y. is a scientific advisor to AlzChem and Amberstone Biosciences and a co-founder, stockholder, and advisory board member of Appia Bio. None of the declared companies contributed to or directed any of the writing of this manuscript.
